# Endogenous IL-27 during toxoplasmosis limits early monocyte responses and their inflammatory activation by pathological T cells

**DOI:** 10.1128/mbio.00083-24

**Published:** 2024-02-20

**Authors:** Daniel L. Aldridge, Devapregasan Moodley, Jeongho Park, Anthony T. Phan, Matthew Rausch, Kerry F. White, Yue Ren, Karin Golin, Enrico Radaelli, Ross Kedl, Pamela M. Holland, Jonathan Hill, Christopher A. Hunter

**Affiliations:** 1 University of Pennsylvania School of Veterinary Medicine, Philadelphia, Pennsylvania, USA; 2 Abata Therapeutics, Waltham, Massachusetts, USA; 3 Kangwon National University College of Veterinary Medicine and Institute of Veterinary Science, Chuncheon, South Korea; 4 Multidimensional Genomics Research Center, Kangwon National University, Chuncheon, South Korea; 5 Surface Oncology, Cambridge, Massachusetts, USA; 6 Comparative Pathology Core, School of Veterinary Medicine, University of Pennsylvania, Philadelphia, Pennsylvania, USA; 7 University of Colorado, Anschuitz Medical Campus, Aurora, Colorado, USA; 8 InduPro, Seattle, Washington, USA; Cornell University, Ithaca, New York, USA

**Keywords:** IL-27p28, toxoplasma, immunity, neutralizing antibody, inflammation

## Abstract

Mice that lack the genes for IL-27, or the IL-27 receptor, and infected with *Toxoplasma gondii* develop T cell-mediated pathology. Here, studies were performed to determine the impact of endogenous IL-27 on the immune response to *T. gondii* in wild-type (WT) mice. Analysis of infected mice revealed the early production of IL-27p28 by a subset of Ly6C^hi^, inflammatory monocytes, and sustained IL-27p28 production at sites of acute and chronic infection. Administration of anti-IL-27p28 prior to infection resulted in an early (day 5) increase in levels of macrophage and granulocyte activation, as well as enhanced effector T cell responses, as measured by both cellularity, cytokine production, and transcriptional profiling. This enhanced acute response led to immune pathology, while blockade during the chronic phase of infection resulted in enhanced T cell responses but no systemic pathology. In the absence of IL-27, the enhanced monocyte responses observed at day 10 were a secondary consequence of activated CD4^+^ T cells. Thus, in WT mice, IL-27 has distinct suppressive effects that impact innate and adaptive immunity during different phases of this infection.

**IMPORTANCE:**

The molecule IL-27 is critical in limiting the immune response to the parasite *Toxoplasma gondii*. In the absence of IL-27, a lethal, overactive immune response develops during infection. However, when exactly in the course of infection this molecule is needed was unclear. By selectively inhibiting IL-27 during this parasitic infection, we discovered that IL-27 was only needed during, but not prior to, infection. Additionally, IL-27 is only needed in the active areas in which the parasite is replicating. Finally, our work found that a previously unstudied cell type, monocytes, was regulated by IL-27, which contributes further to our understanding of the regulatory networks established by this molecule.

## INTRODUCTION

IL-27 is a heterodimeric cytokine, composed of the IL-27p28 and EBI3 sub-units, and signals through a receptor composed of gp130 and the IL-27Rα (1, 2). Initial studies have focused on the ability of IL-27 to promote T cell responses, and there are examples where adjuvant-induced production of IL-27 is required for the expansion of vaccination-generated CD8^+^ T cells (3, 4). However, in multiple models of infection, such as *Toxoplasma gondii*, *Trypanosoma cruzi*, malaria, and helminth infection, mice that lack the IL-27Rα or individual IL-27 sub-units develop enhanced CD4^+^ and CD8^+^ T cell responses that result in exaggerated disease (5–9). While IL-27 has profound regulatory effects on effector T cell responses, the majority of *in vivo* studies on the biology of IL-27 have employed mice that lack the IL-27R or the individual cytokine sub-units (1, 6, 8). Consequently, it has been a challenge to distinguish the effects of IL-27 on developing versus established T cell responses. Thus, there are questions about whether IL-27 constrains the initial development of pathological T cell responses and/or acts at sites of inflammation to limit pathological effector responses. Although there are genetic approaches to perform lineage-specific deletion of IL-27 components (10–12), the use of exogenous antagonists of IL-27 provides an opportunity to address how endogenous IL-27 in a wild-type (WT) setting impacts different phases of the immune response (13).

Murine models of toxoplasmosis have provided insights into the pathways that influence the development of the cell-mediated immunity required for resistance to an intracellular infection. For example, the innate-immune production of IL-12 promotes the development of parasite-specific CD4^+^ and CD8^+^ T cells, which, in turn, produce IFN-γ that limits parasite replication (14). This experimental system has also proven useful to understand the ability of IL-27 to limit pathological T cell responses. Thus, in the absence of IL-27, mice infected with *T. gondii* develop a CD4^+^ T cell-dependent immune pathology characterized by elevated production of IFN-γ, IL-17, and TNFα (8, 15). While these studies have focused on the suppressive effects of IL-27 on T cell responses, they have not addressed the possible impact of IL-27 on innate responses to *T. gondii* or distinguished the impact of endogenous IL-27 at different stages of infection.

In this study, systemic levels of IL-27p28 correlated with the course of infection, and use of the IL-27p28 reporter mouse highlighted inflammatory monocytes as the major source of this cytokine. Similar to studies with IL-27 deficient mice, the administration of a well-validated anti-IL-27p28 neutralizing antibody prior to, and throughout, infection resulted in enhanced T cell responses and the development of immune pathology. Unexpectedly, transcriptional profiling of splenocytes from anti-IL-27 treated mice revealed that blockade also resulted in enhanced monocyte and neutrophil responses by day 5 of infection; however, amplification of these responses later in infection was dependent on CD4^+^ T cells. Additional analysis of parasite-specific T cells from mice treated with anti-IL-27p28 treatment indicated that IL-27 could restrain effector cell expansion. Together, these studies establish that endogenous IL-27 has suppressive effects that impact the crosstalk between innate and adaptive immunity that define the balance between protective and pathological responses during infection with *T. gondii*.

## MATERIALS AND METHODS

### Mice

Female C57BL/6 mice were purchased (~6-week-old) from Taconic labs. IL-27-p28-GFP reporters (3) were housed and bred in specific pathogen-free (SPF) facilities in the Department of Pathobiology at the University of Pennsylvania in accordance with institutional guidelines (IACUC# 805045). Cysts of ME49 strain of *T. gondii* were collected from chronically infected CBA/ca mice brain tissues. Then, experimental mice were infected i.p. with 20 cysts. Anti-IL-27p28 antibody was injected i.p. (1 mg/mouse) at −3, 0, 4, and 7 dpi or 0, 4, and 7 dpi for analysis of the acute phase of infection, with the same dose of isotype IgG given to control mice. In chronic infection, the same amounts of antibodies were administrated but now at days 20, 24, 28, and 32 post infection.

### Anti-IL-27p28 antibody

**Fig 1:**
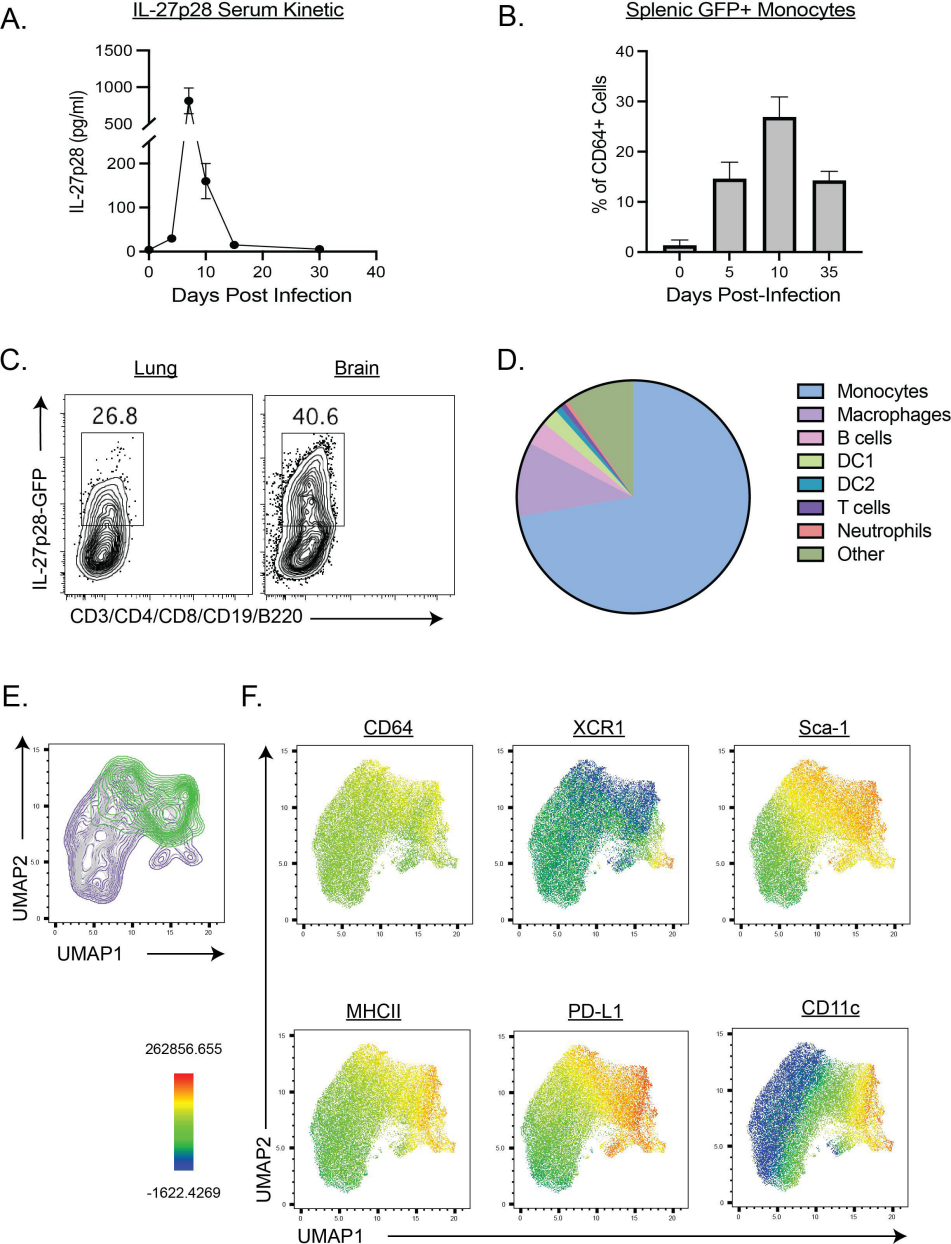
IL-27p28 expression in *T. gondii* infection. (**A**) Kinetics of circulating IL-27p28 during toxoplasmosis. (**B and C**) Expression of IL-27p28 was determined among CD64^+^ monocytes and macrophages at indicated dates of post infection. (**D**) Cell type expression of IL-27p28 in the spleen of infected mice at 5 dpi was determined by flow cytometry. (**E and F**) 10,000 monocyte events were pooled from 3 infected reporter mice, for 30,000 total events. Unsupervised UMAP analysis was performed based on the expression of 15 surface markers (see Materials and Methods), excluding IL-27p28 GFP. Supervised gating of IL-27p28-GFP^+^ and GFP^–^ cells was then overlayed over the UMAP output (**E**). (**F**) Expression of six surface markers most highly associated with differences between GFP^+^ and GFP^–^ clusters are shown.

The human monoclonal antibody (SRF381) specific for IL-27p28 was provided by Surface Oncology and has been described previously (16). This is a fully human IgG1 that binds to the p28 subunit of IL-27 (Fig. S1A), blocks murine IL-27-mediated STAT1 phosphorylation in murine splenic CD3^+^ T cells (Fig. S1B), inhibits murine IL-27-induced PD-L1 expression on splenic CD8^+^ T cells (Fig. S1C), and leads to antibody-mediated accumulation of murine IL-27 p28/IL-30 in the plasma of mice (Fig. 1D).

SRF381 binding to recombinant protein: murine IL-27, human IL-27, murine p28, human EBI3, human IL-12, or human IL-23 (R&D Systems, Minneapolis, MN) were coated on MSD QuickPlex plates (Meso Scale Discovery, Rockville, MD) at 0.5 µg/mL and incubated overnight at 4°C. The plate was blocked with PBS/BSA/Tween20. SRF381 was added at 0.5 µg/mL and incubated for 2 h at room temperature. Antibody binding was detected with goat anti-human antibody sulfo-tag antibody (Meso Scale Discovery).

SRF381-mediated inhibition of recombinant murine IL-27 induced STAT1 phosphorylation in splenic CD3^+^ T cells: murine splenocytes were preincubated with SRF381 at concentrations ranging from 20 µg/mL to 3 ng/mL for 1 h. Recombinant murine IL-27 (20 ng/mL; R&D Systems) was added, and cells were incubated for 30 min. Cells incubated with mIL-27 alone served as the 0% inhibition control, and cells incubated with PBS served as the 100% inhibition control. Cells were stained with FITC-anti-CD3 and then fixed and permeabilized in BD Phosflow Lyse/Fix Buffer according to the manufacturer’s instructions (BD Biosciences, San Jose, CA) before staining with PE-pSTAT1 (pY701) (BD Biosciences). pSTAT1 staining was identified in CD3^+^ splenic T cells using an LSRFortessa X-20 flow cytometer (BD Biosciences), and percent inhibition was calculated.

Hydrodynamic transfection of IL-27 minicircles and *in vivo* blockade of PD-L1 expression with SRF381: 6-week-old female Balb/c mice were injected with 20 µg of either empty vector or linked murine IL-27 minicircle DNA (System Biosciences, Palo Alto, CA) in 2 mL 0.9% normal saline via the tail vein over the course of 5 s. Injected animals were transferred to an empty cage with a heating pad to recover for 5 min. Whole blood was collected into K2-EDTA tubes for plasma separation 24 h after minicircle injection, and plasma IL-27 levels were confirmed by ELISA. Five days after minicircle injection, mice were treated IP with 1,000 µg SRF381 or human IgG1 isotype control antibodies. Animals were sacrificed 3 days after antibody treatment, and spleens were collected. Single-cell splenocyte suspensions were prepared by mechanical dissociation followed by red blood cell lysis in ACK buffer. FcγR II/III was blocked by preincubating cells with rat anti-mouse CD16/CD32 mAb (1 µg per million cells; Biolegend, San Diego, CA) in PBS with 2% FBS and 2 mM EDTA. Cells were stained with APC-, Brilliant Violet 510-, and Brilliant Violet 711-conjugated mAbs against murine CD4 (clone GK1.5), CD8 (53–6.7), and PD-L1 (10F.9G2) (Biolegend). Cell-associated fluorescence was measured using an LSRFortessa X-20 flow cytometer (BD Biosciences), and analysis was performed using FlowJo software (Tree Star, Ashland, OR).

Plasma detection of murine IL-27 p28/IL-30 after SRF381 administration: 6-week-old female C57BL/6 mice were injected with 1 mg of SRF381 or hIgG1 antibodies intravenously. Blood was collected at the indicated timepoints, and plasma was prepared and frozen at −80°C until testing. Plasma levels of p28 were determined using the murine IL-27 p28/IL-30 antibody pairs from R&D systems by MSD.

### Cell staining and flow cytometry

Single-cell splenocyte suspensions were prepared by mechanical dissociation followed by red blood cell lysis in ACK buffer. FcγRII/III was blocked by preincubating cells with rat anti-mouse CD16/CD32 mAb (1 µg per million cells; Biolegend, San Diego, CA) in PBS with 2% FBS and 2 mM EDTA. Cells were stained with APC-, Brilliant Violet 510-, and Brilliant Violet 711-conjugated mAbs against murine CD4 (clone GK1.5), CD8 (53–6.7), and PD-L1 (10F.9G2) (Biolegend). Cell-associated fluorescence was measured using an LSRFortessa X-20 flow cytometer (BD Biosciences), and analysis was performed using FlowJo software (Tree Star, Ashland, OR).

Cells from indicated organs were prepared as described (17) and were stained with LIVE/DEAD Fixable Aqua Dead Cell Stain (L3457; Thermo Fisher) and antibodies specific for CD4 (GK1.5, 100447; BioLegend), CD8 (53–6.7, 562283; BD Biosciences), LFA1 (H155-78, 141008; BioLegend), CD11a (M17/4, 101124; BioLegend), KLRG1 (2F1, 11-583-82, eBiosciences), CXCR3 (CXCR3-173, 126516; BioLegend), Thy1.1/CD90.1 (HIS51, 47-0900-82; Invitrogen), MHC class I-SVLAFRRL, MHC class II (MHC II)-AVEIHRPVPGTAPPSFSS, Ly6G (1A8, 2343097; BD), CD11c (N418, 749039; BD), Sca-1 (D7, 45-5981-82; Invitrogen), CD11b (M1/70, 101257; BioLegend), XCR1 (ZET, 148220; BioLegend), MHC II/I-A/I-E (M5/114.15.2), Ly6C (HK1.4, 128041; BioLegend), CD64/FcγRI (X54-5/7.1, 139314; BioLegend), TNFα (MP6-XT22, 506349; BioLegend), PD-L1/CD274 (MIH5, 12-5982-82; Invitrogen). Tetramers were obtained from the National Institutes of Health Tetramer Core Facility.

### ELISAs

For IFN-γ measurement, splenocytes (3 × 10^5^ per well) were cultured for 72 h with soluble Toxoplasma antigen (STag, 12.5 ng/mL). Serum and supernatant from STag-stimulated splenocytes were collected and IFN-γ ELISA was performed with anti-IFN-γ antibody clones AN18 and R4-6A2 for capture and detection, respectively. For IL-27p28 detection, mouse IL-27p28/IL-30 Quantikine ELISA kit (M2728; R&D Systems) was used according to the manufacturer’s protocol.

### Histology

Brain tissues were fixed in 10% buffered formalin and 7 µm paraffin-sections were prepared. Sections were stained with hematoxylin and eosin (H&E) and slides were visualized on an LSM-510 Meta confocal microscope (Zeiss). Formalin-fixed liver samples were processed for paraffin embedding, sectioned at 5 mm, and stained with hematoxylin and eosin (HE). The resulting slides were analyzed by a board-certified veterinary pathologist blinded to experimental design, and the following parameters were scored: hepatic necrosis, endophlebitis, thrombosis, and inflammatory/immune cell infiltrate. Depending on severity and extent of tissue distribution these findings were scored on a 0–2 (hepatic necrosis, endophlebitis, and thrombosis) or 0–3 (inflammatory/immune cell infiltrate) scale with 0 indicating absence of the lesion and 2 (or 3 in the case of inflammatory/immune cell infiltrate) indicating severe changes.

### Gene expression profiling

Mouse splenocytes were prepared by mechanical dissociation of whole spleens, followed by ACK lysis of red blood cells. After staining with antibody cocktails, total CD45^+^ cells (day 5 post-infection; *n* = 5 per treatment) and tetramer-positive T cells (day 10 post-infection; *n* = 5 per treatment) were sorted by FACS directly into lysis buffer. Total RNA was extracted from sorted cells with the RNeasy Mini Kit (Qiagen, Cat. No: 74104) and adjusted to 20 ng/mL in nuclease free water (Qiagen, Cat. No: 19101). Gene expression profiling was performed on Affymetrix GeneChip Mouse Gene 2.0 ST Arrays (Applied Biosystems, Cat. No: 902118). Processing of RNA samples, hybridization, and array scanning were carried out using standard Affymetrix GeneChipTM protocols at the Boston University Microarray and Sequencing Resource (BUMSR). All CEL files were normalized by Robust Multi-array Average (RMA) (18), and gene expression data were preprocessed by removing unexpressed probes and discarding transcripts with high inter-replicate coefficient of variance. Subsequent analyses (mean expression, fold change, *t* test) were performed in R. Data sets are available at the National Center for Biotechnology Information Gene Expression Omnibus under accession number GSE184350.

### Statistics

An unpaired Student’s *t*-test, Welch’s *t*-test, nonparametric Mann-Whitney *U*-test, and Log-rank test were used to determine the significance of differences unless otherwise indicated; *P* values of less than 0.05 were considered significant.

## RESULTS

### Analysis of infection-induced IL-27

To determine the kinetics and cellular source of IL-27p28 during toxoplasmosis, C57BL/6 mice were infected with *T. gondii* and serum levels of IL-27p28 were measured by ELISA. In uninfected mice, there were low serum levels of IL-27p28, but after infection, there was an increase in systemic IL-27p28 at 4 dpi, a peak at 7 dpi, and a return to baseline by 15 dpi (Fig. 1A). To assess the cellular source of IL-27, IL-27p28-GFP reporter mice (3) were infected and flow cytometry used to compare patterns of expression in naïve and infected mice. In the spleen of uninfected mice, neutrophils, DCs, and T cells did not express significant levels of GFP (data not shown), but a small population of GFP^+^ monocytes (CD11b^+^CD64^+^F4/80^+^) were detected. By 5 dpi, p28-GFP^+^ monocytes had increased, peaked at 10 dpi but remained elevated at 35 dpi (Fig. 1B). At 35 dpi, the lungs and the brains are sites of ongoing infection and inflammation. Consistent with this, there was substantial expression of IL-27p28 at this timepoint from inflammatory monocytes and macrophages in these sites (Fig. 1C).

At 5 dpi, splenocytes from the p28-GFP reporters were analyzed by flow cytometry to determine the cellular sources of IL-27. In these studies, several cell types were p28-GFP^+^, but monocytes and macrophages were the overwhelmingly dominant sources of IL-27 (Fig. 1D). Analysis of CD11b^+^Ly6C^+^ monocytes from these mice was then performed using high-dimensional flow cytometry of multiple surface markers (see Materials and Methods) followed by dimensional reduction via UMAP, excluding p28-GFP (Fig. 1E). In Fig. 1E, the whole monocyte population is shown, with the expression of p28-GFP illustrated in green. This specific cluster of IL-27p28^+^ monocytes was characterized by heighted expression of CD64, Sca-1, MHCII, and PD-L1. Conversely, it showed reduced expression of XCR1 in comparison to the IL-27p28^−^ monocyte population. These data indicate that activated monocytes and macrophages are the dominant source of IL-27, and it is produced at sites of infection during both the acute and chronic phases.

### Impact of anti-IL-27p28 antibody on acute infection

To determine the impact of timed inhibition of IL-27 on infection-induced immune responses, a monoclonal antibody specific for IL-27p28 (SRF 381) was utilized. This human IgG1 binds to the p28 subunit of human and mouse IL-27 (Fig. S1A) and blocked the ability of murine IL-27 to induce STAT1 phosphorylation and PD-L1 expression in murine splenic CD3^+^ T cells with an IC50 of approximately 440 ng/mL (Fig. S1B and C). In naïve, C57BL/6 mice, treatment with the isotype control did not alter the basal levels of IL-27p28, but administration of anti-IL-27p28 resulted in increased plasma levels of IL-27-p28 within 24 h, plateauing by days 7–14 (Fig. S1D). These data indicate that antibody-mediated cytokine neutralization and accumulation of the antibody-cytokine complex in plasma takes 3–4 days to reach steady state, a pharmacodynamic property observed with other anti-cytokine antibodies (19, 20).

**Fig 2:**
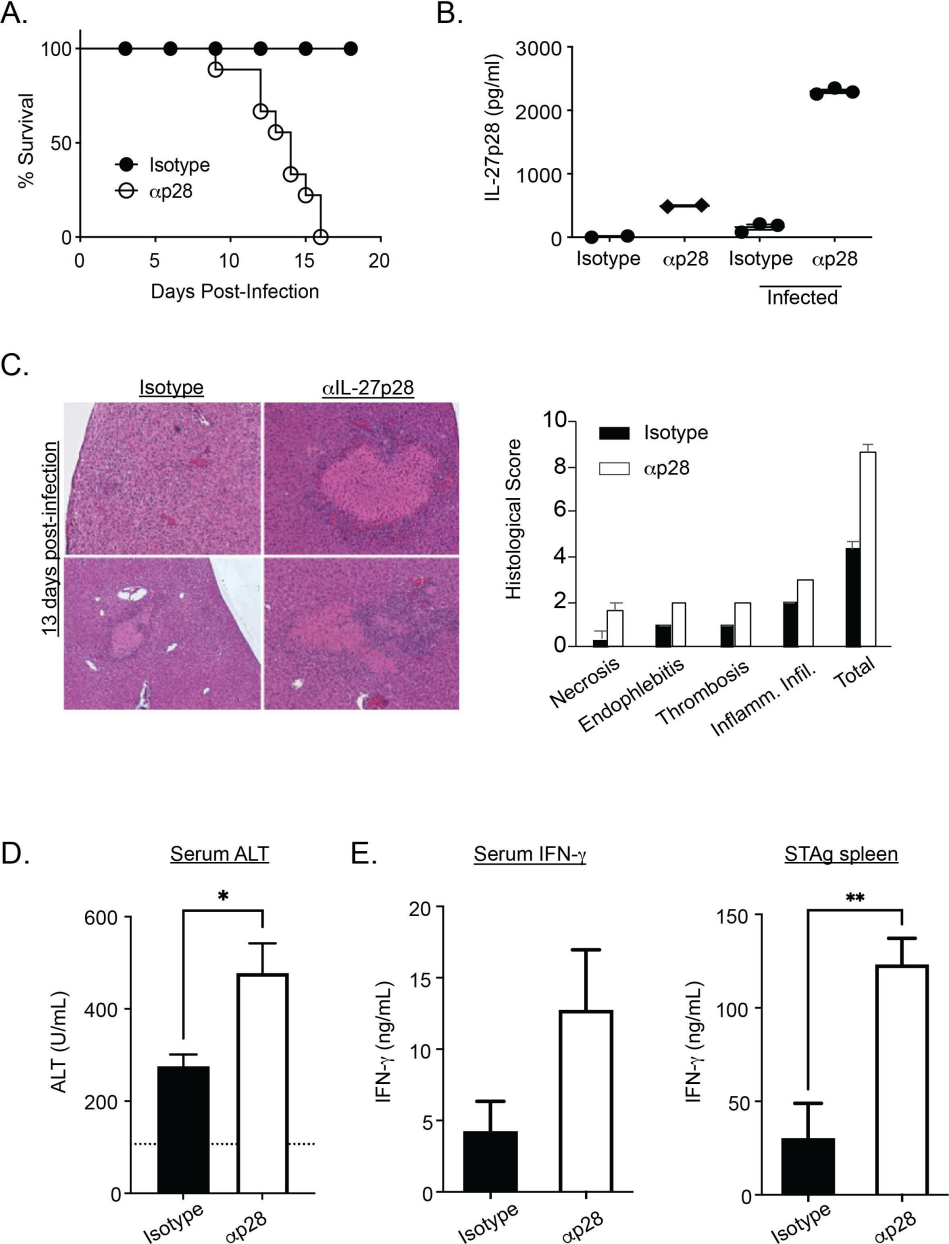
IL-27p28 neutralization results in pathology similar to p28 deficiency in acute toxoplasmosis. (**A**) Survival of IgG isotype controls (*n* = 10) and anti-IL-27p28 treated mice (*n* = 10) with *T. gondii* infection. (**B**) Serum levels of IL-27p28 were measured in naïve (left two bars) and infected (right two bars) with isotype or blockade treatment. Liver histology (**C**) and serum ALT (**D**) in indicated groups at 10 days post infection. (**E**) IFN-γ level in the serum (left) or from splenocytes incubated with *T.gondii* antigen (right) at 10 dpi were determined by ELISA (*n* = 5–8, from 2 to 3 experiments, mean ± SEM). * and ** indicate *P* ≤ 0.05 and 0.01, respectively.

When C57BL/6 mice were treated with isotype or anti-IL-27p28 starting on −3 dpi and then treated every 3–4 days thereafter, those that received the isotype control survived infection, whereas those given anti-IL-27p28 succumbed to this challenge by 15–20 dpi (Fig. 2A), kinetics that are similar to IL-27R-deficient mice. In infected mice, the treatment was associated with a marked increase in circulating levels of IL-27p28 (Fig. 2B) indicative of elevated cytokine production and antibody-mediated blockade. Histological analysis revealed that while infected mice had areas of inflammation in the liver, neutralization of IL-27p28 resulted in increased levels of hepatic damage characterized by enhanced necrosis, endophlebitis, thrombosis, and inflammatory cell accumulation (Fig. 2C). This was associated with elevated serum levels of alanine aminotransferase (ALT), a marker of hepatic damage (21) (Fig. 2D). The neutralization of IL-27 also resulted in elevated circulating levels of IFN-γ as well as increased *ex vivo* production of IFN-γ by splenocytes stimulated with STAg (Fig. 2E). Thus, in mice infected with *T. gondii*, the blockade of endogenous IL-27p28 recapitulates the increased production of IFN-γ and pathology observed in IL-27R and IL-27p28 KO mice (8, 17, 22).

### Impact of IL-27p28 neutralization on innate immune responses to acute toxoplasmosis

**Fig 3:**
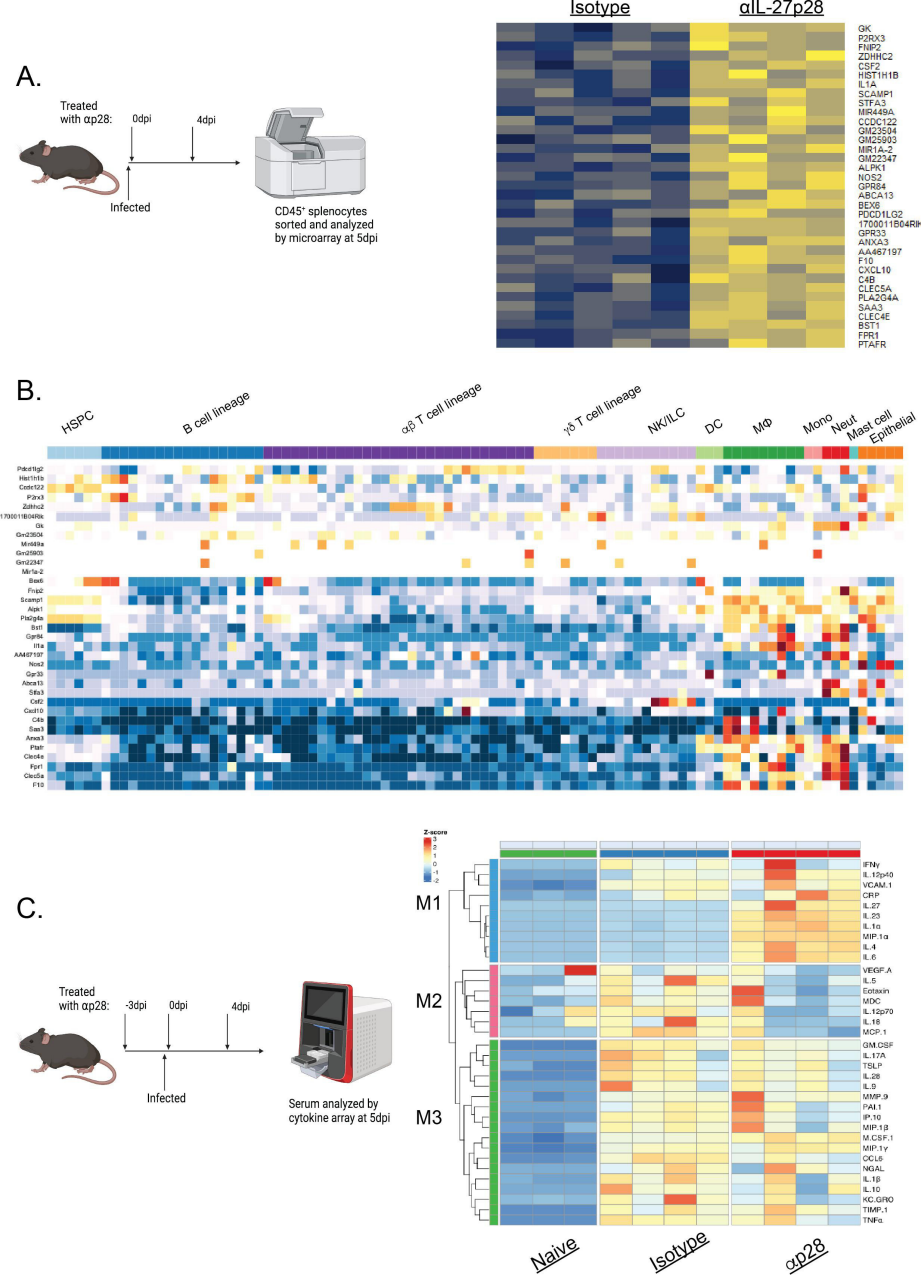
IL-27p28 blockade results in differential transcript and cytokine expression profiles at 5 dpi. (**A**) A schematic of IL-27 blockade is shown (left). Gene expression between isotype and αIL-27p28 treated CD45^+^ cells was then compared, with the top 35 differentially expressed genes shown. (**B**) CIBERSORT (23), a bioinformatics technique that identifies cell types based on gene expression profiles, assigned immune cell types to the profile identified in (**A**). Warmer colors indicate greater association with the profile, while cooler colors indicate less association. (**C**) Serum from naive, isotype, or anti-IL-27p28 treated mice was collected at 5 dpi and analyzed by multiplexed cytokine analysis. Unbiased hierarchical clustering was then performed, and three dominant clusters are shown.

To assess the impact of IL-27 neutralization on the innate immune response to *T. gondii,* an unbiased transcriptional profiling approach was utilized. WT mice were infected and treated with either isotype control IgG or anti-IL-27p28 on 0 dpi and 4 dpi (Fig. 3A). At 5 dpi, splenocytes that express the transmembrane protein CD45 (expressed by cells of hematopoietic origin) were sort purified from isotype control and anti-IL-27p28-treated mice, transcriptionally profiled by microarray, and analyzed for differential gene expression. The 35 most differentially expressed genes are shown (Fig. 3A). Many of these genes corresponded to innate immune cell activation (e.g., *CSF2* and *CLEC5A*) as well as IFN-γ activation of innate cells (*CXCL10*, *NOS2*, and *PDCD1LG2*). To identify which cells were altered due to this early IL-27 blockade, these differentially expressed genes were used to probe transcriptional signatures from the Immunological Genome Project (ImmGen; data set names shown in Table 1) using the MyGeneset tool to assign immune cell types to the profile identified in Fig. 3A. This analysis revealed that neutralization of IL-27 resulted in the emergence of an early signature most prominently associated with activated macrophages, monocytes, and neutrophils (Fig. 3B).

**TABLE 1:** ImmGen gene signature labels for groups shown in Fig. 3B

Group identifier	ImmGen identifier
HSPC	LTHSC_34−_BM
	LTHSC_34+_BM
	STHSC_150−_BM
	MMP2_150+48+_BM
	MMP3_48+_BM
	MMP4_135+_BM
B cell lineage	proB_CLP_BM
	proB_FrA_BM
	proB_FrBC_BM
	proB_FrD_BM
	B_FrE_BM
	B_T1_Sp
	B_T2_Sp
	B_T3_Sp
	B_Sp
	B_Fem_Sp
	B_Fo_Sp
	B_MZ_Sp
	B_mem_Sp
	B_GC_CC_Sp
	B_GC_CB_Sp
	B_PB_Sp
	B_PC_Sp
	B_PC_BM
	B1b_PC
ab T cell lineage	preT_DN1_Th
	preT_DN2a_Th
	preT_DN2b_Th
	preT_DN3_Th
	T_DN4_Th
	T_ISP_Th
	T_DP_Th
	T_4_Th
	T_4_Nve_Sp
	T_4_Nve_Fem_Sp
	T_4_Sp_aCD3+CD40_18 h
	T_4_19-8-TCRb+_Sp
	T_8_Th
	T_8_Nve_Sp
	T8_TN_P14_Sp
	T8_TE_LCMV_d7_Sp
	T8_MP_LCMV_d7_Sp
	T8_IEL_LCMV_d7_Gut
	T8_Tcm_LCMV_d180_Sp
	T8_Tem_LCMV_d180_Sp
	T8_IEL_LCMV_d32_SI
	T8_LCMV_d32_Bl
	T8_LCMV_d32_Fat
	T8_LCMV_d32_Kd
	T8_LCMV_d32_Lv
	T8_LCMV_d32_SG
	T8_LCMV_d32_Sp
	Treg_4_25hi_Sp
	Treg_4_FP3+_Nrplo_Co
	NKT_Sp
	NKT_Sp_LPS_3 h
	NKT_Sp_LPS_18 h
	NKT_Sp_LPS_3d
	NKT_19–8-TCRb+ CD1daGalCerTet+_Lu
	NKT_19–8-TCRb+ CD1daGalCerTet+_Sp
	NKT_19–8-TCRb+ CD1daGalCerTet+_Th
	NKT_19–8-TCRb+ CD1daGalCerTet+_Lv
gd T cell lineage	Tgd_g2+d17_24a+_Th
	Tgd_g2+d1_24a+_Th
	Tgd_g1_1+d1_24a+_Th
	Tgd_g2+d17_LN
	Tgd_g2+d1_LN
	Tgd_g1_1+d1_LN
	Tgd_Sp
NK/ILC	NK_27–11b+_Sp
	NK_27+11b−_Sp
	NK_27+11b+_Sp
	NK_27–11b+_BM
	NK_27+11b−_BM
	NK_27+11b+_BM
	ILC2_SI
	ILC2_ST2−_SI
	ILC3_NKp46-CCR6−_SI
	ILC3_CCR6+_SI
	ILC3_NKp46+_SI
DC	DC_4+_Sp
	DC_8+_Sp
	DC_pDC_Sp
Macrophage	MF_PC
	MF_Fem_PC
	MF_226+II+480lo_PC
	MF_102+480+_PC
	MF_RP_Sp
	MF_Alv_Lu
	MF_pIC_Alv_Lu
	MF_microglia_CNS
	MF_AT
Monocyte	Mo_6C+II−_Bl
	Mo_6C-II−_Bl
Neutrophil	GN_BM
	GN_Sp
	GN_Thio_PC
Mast cell	MC_heparinase_PC
	Ep_MEChi_Th
	FRC_CD140a+_Madcam-_CD35-_SLN
	LEC_SLN
	BEC_SLN
	IAP_SLN

To compliment this approach, a multiplexed cytokine array was used to assess the impact of IL-27 neutralization on serum cytokine levels at 5 dpi (Fig. 3C). Here, IL-27 was neutralized at days −3, 0, and 4 dpi before serum was collected at 5 dpi. Hierarchical clustering analyses indicated three dominant modules (M1-3) of cytokine expression apparent in these mice. Infection induced a shared module, M3, that was seen only in infected mice, contained M-CSF, IL-1β, and TNFα, and was not affected by IL-27 neutralization (Fig. 3C). IL-27 blockade, however, resulted in a module, M1, composed of factors that were either absent in infected serum (IL-1a, MIP, IL-4, IL-6, IL-23) or induced at low levels but were increased following IL-27 neutralization (IFN-γ, IL-12p40, VCAM-1, and CRP). Previous studies have shown this is a time point when serum levels of IFN-γ in WT and IL-27 KO mice are similar, but these data sets highlight that in at least one mouse treated with αIL-27p28 that this has started to diverge. This module also contained IL-27 (with anti-IL-27p28 stabilizing the protein) as a positive control. Finally, M2 (which contained VEGF, IL-5, eotaxin, IL-18, IL-12, and MCP-1) was expressed highly in isotype-treated samples but was reduced after IL-27 neutralization. These latter changes suggest that these cytokines are dependent (directly or indirectly) on the production of IL-27. Thus, early loss of IL-27 signaling during toxoplasmosis has a marked impact on the initial immune responses to this infection.

### IL-27 regulates monocyte responses to acute toxoplasmosis

**Fig 4:**
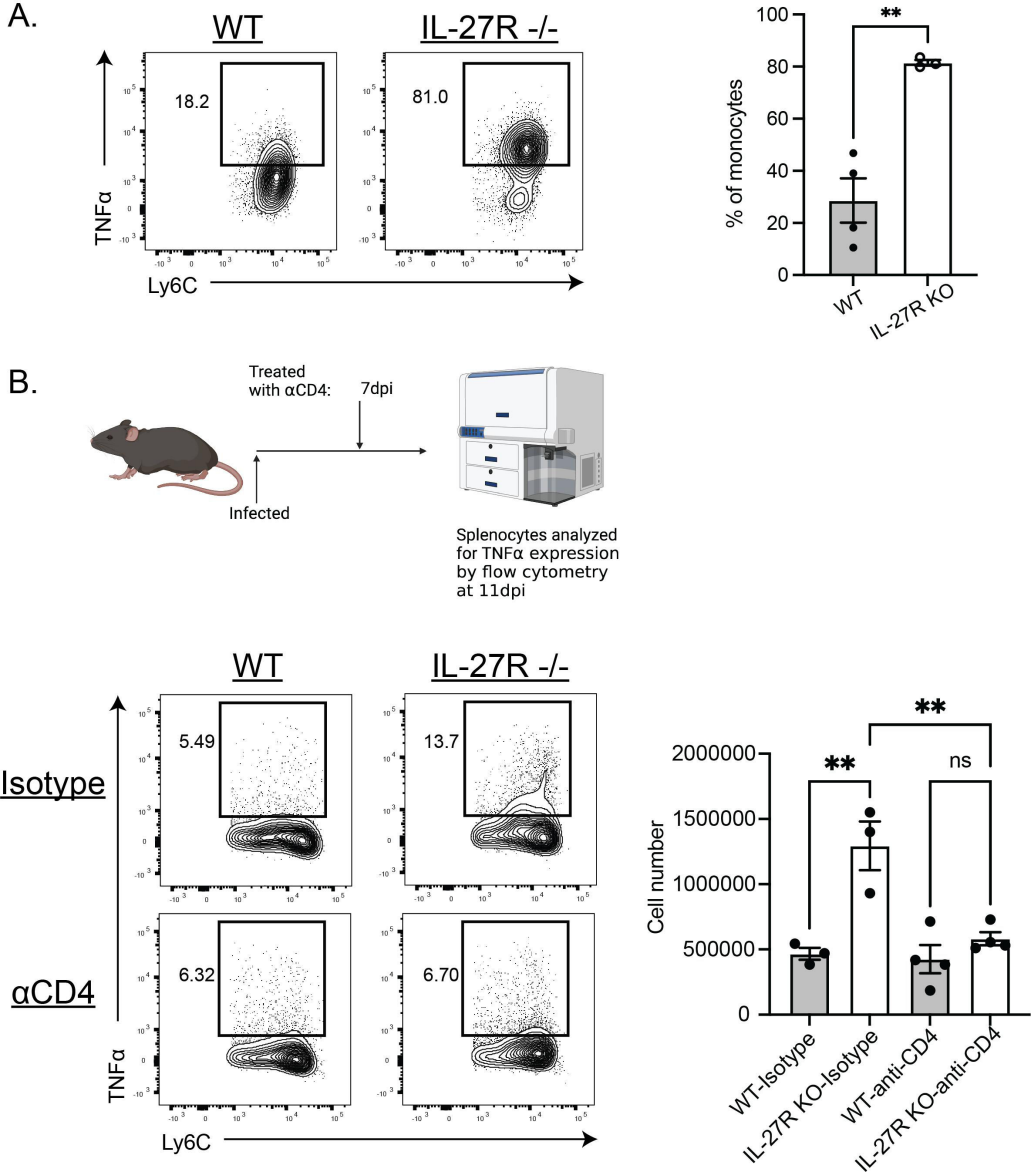
Inflammatory monocyte responses are enhanced in the absence of IL-27 and impacted by CD4^+^ T cell responses. (**A**) Splenocytes from WT and IL-27R^−/−^ mice at 10 dpi were isolated, incubated with BFA and GolgiStop for 4 h before analyzing monocyte expression of TNFα by flow cytometry. Representative plots are shown (left) and quantified (right). Statistical analysis was performed using Welch’s *t*-test. ** indicates *P* ≤ 0.01. (**B**) CD4^+^ T cells were depleted from WT and IL-27R KO mice at 7 dpi. Splenocytes were then isolated at 11 dpi, and monocyte TNFα expression was analyzed as above. Representative flow plots are shown (left) and quantified (right). Statistical analysis was performed using one-way ANOVA, followed by Tukey’s multiple comparison test. *, **, and **** indicate *P* ≤ 0.05, 0.01, and 0.0001, respectively.

The enhanced monocyte transcriptional signature and systemic cytokines (Fig. 3B and C) with IL-27 blockade were reminiscent of sepsis. Indeed, a comparison of a publicly accessible data set of human septic monocytes (24) showed that greater than 50% of genes associated with the “MS1” and “MS2” septic monocyte phenotypes were induced by blockade of IL-27 at 5 dpi (Fig. S2A). To determine if the enhanced early monocyte responses observed with αIL-27p28 were also apparent in IL-27 deficient mice, studies were performed with mice that lacked the IL-27 EBI3 subunit (Fig. S2B) or IL-27Rα (Fig. 4A), and their monocytes responses analyzed at 5 or 10 dpi, respectively. At both time points, there was a significant increase in TNFα producing monocytes (Fig. 4A; Fig. S2B). As mature monocytes do not express the IL-27R (Fig. S2C), experiments were performed to assess the contribution of CD4^+^ T cells to this enhanced monocyte activity. At 7 dpi, CD4^+^ T cells were depleted from infected IL-27R^−/−^ mice, and monocyte responses were then analyzed at 11 dpi (Fig. 4B). IL-27R-deficient mice treated with isotype control antibody showed enhanced monocyte TNFα production in the absence of IL-27 signaling, but this activity was reduced when CD4^+^ T cells were depleted (Fig. 4B). Together, these data indicate that after IL-27 blockade or in gene deficient mice there is an early impact on monocyte responses, but at the peak of inflammation, this is dependent on CD4^+^ T cells.

### IL-27p28 neutralization promotes T cell activity during acute and chronic toxoplasmosis

**Fig 5:**
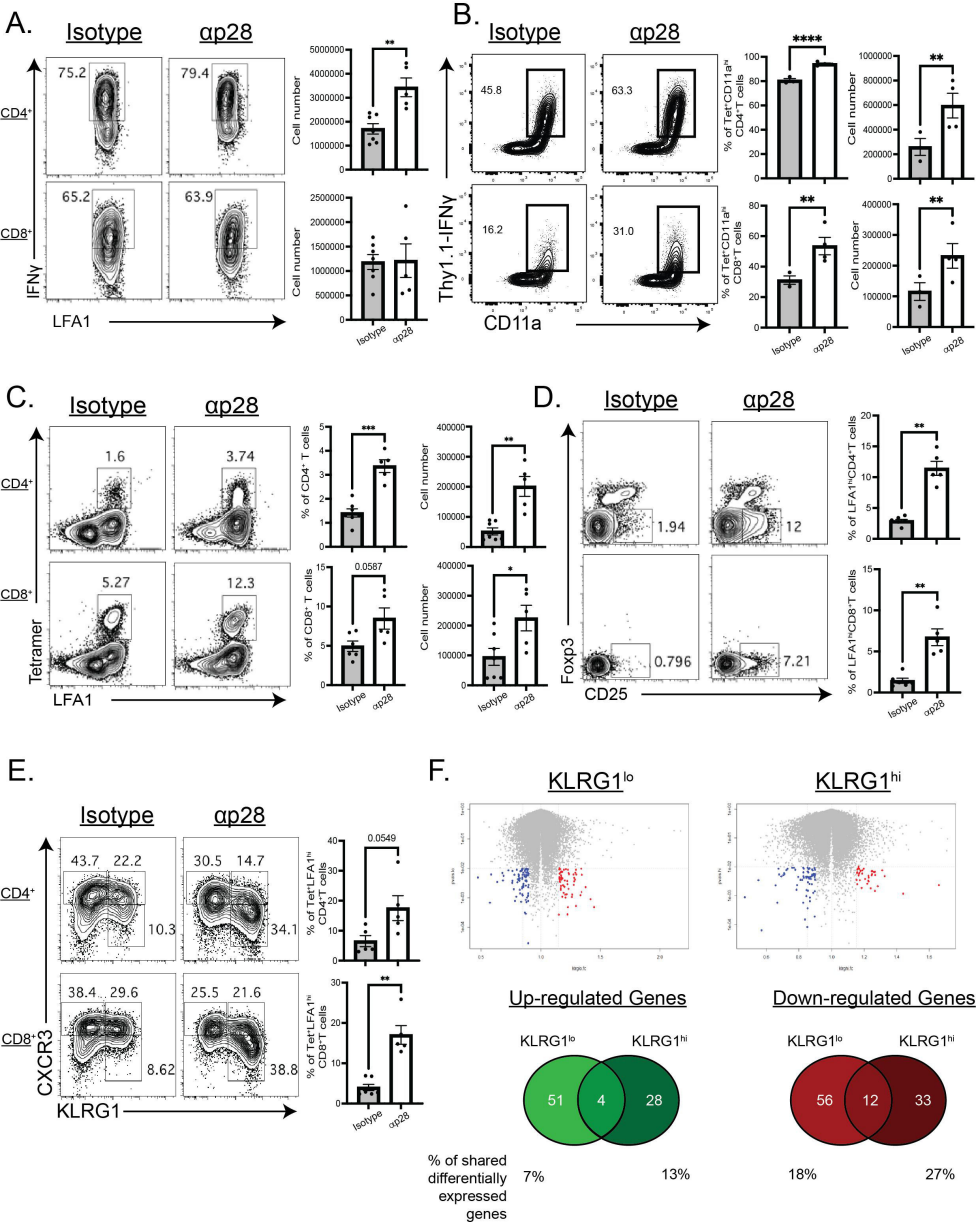
T cell responses to anti-IL-27p28 treatment in acute toxoplasmosis. (**A**) *T. gondii*-specific CD4^+^ (top) and CD8^+^ (bottom) T cells from the spleens of mice treated with either isotype (left) or anti-IL-27p28 (right) antibodies were analyzed by flow cytometry at 10 dpi for their expression of IFNγ following stimulations with PMA + ionomycin treatment. Representative flow plots are shown (left) and cell numbers quantified (right). (**B**) IFNγ-Thy1.1 reporters were treated with isotype (left) or anti-p28 antibodies (right), infected as above and CD4^+^ (top) and CD8^+^ (bottom) T cells were measured for reporter expression from splenocytes at 10 dpi. Representative flow plots of CD11a^hi^Thy1.1^+^ cells are shown (left) and the percentage and numbers of tetramer^+^ CD11a^hi^Thy1.1^+^ cells quantified (right). (**C**) *T. gondii*-specific and (**D**) polyclonal, Foxp3^−^CD25^+^ CD4^+^ (top) and CD8^+^ (bottom) T cells in the spleens of isotype IgG and anti-IL-27p28 antibody mice at 10 dpi were analyzed. (**E**) Comparison of T cell subset by CXCR3 and KLRG1 expression among *T.gondii* experienced CD4^+^ and CD8^+^ T cells is provided for the indicated groups. (**F**) Microarray analysis of CD4^+^, *T. gondii*-specific KLRG1^lo^ and KLRG1^hi^ cells from splenocytes of mice treated with anti-IL-27p28 or isotype control antibodies was performed. Volcano plots showcasing alterations in gene expression during blockade are shown, with Venn diagrams of the numbers upregulated and downregulated genes in each populations shown below. Representative and combined data collected (mean ± SEM, *n* = 5–9) from 2 to 3 independent experiments. *, **, and **** indicate *P* ≤ 0.05, 0.01, and 0.0001, respectively.

To assess the impact of IL-27p28 neutralization on the pathogen-specific CD4^+^ and CD8^+^ T cell responses, their ability to produce IFN-γ, as well as their phenotype and numbers, was measured during acute infection. While T cells from naïve mice produced little IFN-γ (data not shown), following restimulation of splenocytes from infected mice, there was robust production of IFN-γ by CD4^+^ and CD8^+^ T cells. The numbers of IFN-γ^+^ CD4^+^ T cells, especially, were further elevated in mice treated with anti-IL-27p28 (Fig. 5A). IFN-γ reporter mice (25, 26), which express the surface protein Thy1.1 under the control of the IFN-γ promoter, were then infected and treated with anti-IL-27. CD4^+^ T cells showed the greatest expression of IFN-γ, and the number and proportion of toxoplasma-specific, IFN-γ producing T cells was significantly enhanced during blockade (Fig. 5B). Similarly, *T. gondii*-specific CD8^+^ T cells also showed significantly enhanced IFN-γ production during blockade (Fig. 5B).

The use of Class I and II tetramers to identify pathogen-specific CD4^+^ and CD8^+^ T cells (27, 28) revealed that neutralization of IL-27 prior to, and throughout, infection resulted in an increased number of both of these populations in the spleen (Fig. 5C). In addition, gating on the polyclonal, LFA1^hi^ effector CD4^+^ and CD8^+^ T cells showed that in the absence of IL-27 there was enhanced expression of CD25, the high-affinity IL-2Rα chain (Fig. 5D). The inclusion of Foxp3 in these plots allows the identification of Treg cells, but this treatment did not appear to significantly alter frequency or number of these lymphocytes (data not shown).

The combination of the surface molecules KLRG1 and CXCR3 has been used to identify memory and effector T cell sub-populations during toxoplasmosis infection (29), with CXCR3^+^KLRG1^−^, CXCR3^+^KLRG1^+^, and CXCR3^−^KLRG1^+^ representing memory, intermediate, and effector T cell phenotypes, respectively. Analysis of these populations in infected mice revealed that IL-27p28 neutralization resulted in an increased effector, CXCR3^−^KLRG1^+^ cell population (Fig. 5E). To determine if these effects were specific to the acute phase of infection, IL-27 was also neutralized during the chronic phase of infection and again resulted in significantly increased numbers of parasite-specific CD4^+^ and CD8^+^ T cells in the brain (Fig. S3A). Immune infiltration and pathology were also enhanced in the CNS (Fig. S3B), but blockade in the chronic phases of infection did not result in systemic pathology.

Previous studies have established that, in the absence of IL-27, infection-induced pathology is mediated by CD4^+^ T cells. To better understand how IL-27 affects the transcriptional program of parasite-specific CD4^+^ T cells, the class II tetramer was used to sort-purify parasite-specific CD4^+^ KLRG1^lo^ and KLRG1^hi^ populations from infected mice treated with isotype control or anti-IL-27p28. These populations were then used for transcriptional microarray analysis. As expected, the absence of IL-27 signaling resulted in reduced expression of genes previously identified as an IL-27 transcriptional signature in both KLRG1^lo^ and KLRG1^hi^ antigen-specific CD4^+^ T cells (Fig. S4A) (22). Previously, the loss of IL-27 signaling has resulted in the induction of interferon signature genes (ISGs). Treatment with anti-IL-27p28 similarly resulted in the loss of ISG gene signatures in both the KLRG1^lo^ and KLRG1^hi^ T cell populations (Fig. S4A). In both isolated populations, anti-IL-27p28 treatment further led to the enhancement and repression of a variety of genes, with several being shared between both populations (Fig. 5F; Table 2). Additionally, a recent analysis of T cell subset gene signatures and metabolic regulators of memory precursor cells (30) was used to determine if these signatures were upregulated or downregulated during IL-27 blockade (Fig. S4B). In both KLRG1 hi and lo cells, genes associated with an intermediate effector T cell state (T_INT_) were upregulated after IL-27 blockade, while in the KLRG1 lo cells, which contain memory precursors (MP), MP-associated genes were downregulated during blockade. No bias in expression was seen for genes associated with terminally differentiated effector cells (T_E_) or metabolic regulators of MP cell differentiation (data not shown). These data sets indicate that blockade of IL-27 recapitulates the findings at a transcriptional level that have previously been reported in knockout mice*,* and additionally that blockade of IL-27 may drive T cells out of the MP stage and into the T_INT_ stage.

**TABLE 2:** Upregulated and downregulated genes in parasite-specific CD4^+^ KLRG1 hi and lo cells during acute toxoplasmosis with IL-27 blockade^*a*^

	Upregulated genes	Downregulated genes		Upregulated genes	Downregulated genes
KLRG1 lo	CXCR2	STAT1	KLRG1 hi	IL1R2	STAT1
	ANO7	1700019D03RIK		CXCR2	PAPPA2
	GM6104	IKZF2		CXCR1	BC094916
	FAM124B	GM12153		OTOS	IFI204
	PALM	IRGM2		OLFR1384	DRAM1
	ITGA7	GM23341		WDR35	1700020G17RIK
	BC020402	GM5431		GM9257	GM24439
	PRSS57	ABI3		GM22790	2310031A07RIK
	TRIM16	GM11709		EGLN3	IRGM1
	2810001G20RIK	SERPINA3F		TRGJ4	SERPINA3F
	GM6867	RNF144A		FAM213A	GM23084
	TRGJ1	SERPINA9		IFT57	IRF4
	SERPINB9B	ADAMTS6		GM3448	LRRC16A
	ANKRD55	LOC102642243		EMILIN2	IRF9
	GZMA	ZFP459		MS4A6C	OLFR1512
	ITGA2	D630045M09RIK		LRP5	ARHGAP39
	LTB4R1	D830030K20RIK		SESTD1	DCPP1
	FAM213A	ANG		TSPAN18	IGF2R
	GZMC	TRAV5-1		SULF2	GM19500
	PRR5	TRAV8D-2		GM25696	IIGP1
	GM9961	A630038E17RIK		GSTM5	GM4841
	OLFR108	GM6337		SYNPO2	CD274
	—	NR4A1		TMEM64	ANKRD1
	GNAQ	SNTB1		MGLL	GM24640
	ANXA1	ARHGAP39		MTMR10	GM24640
	CERCAM	RTP4		VMN2R49	GM12534
	AA467197	ABCC5		SPIB	IL21
	PRNP	AIRN		DKKL1	1500004A13RIK
	SESTD1	OLFR124		AQP11	GM26355
	WFDC6A	H2-T24		IFITM2	AW011738
	SULF2	IIGP1		MYO1E	GM6297
	SLC39A8	GM4841		GLCE	ISG15
	MND1	GM26183			GBP8
	DHRS3	CD274			GBP4
	GM6460	GRK5			GBP10
	GM8922	GM22323			GM22093
	FLNC	GM23931			SCGB1B3
	IL17RE	MIR669L			VMN2R33
	IGKV4-59	MIR669M-1			ZFP109
	EPS8	SLC43A1			GM15655
	KCNJ8	GM14124			IRF8
	ITGAM	RALGPS1			PLSCR1
	ITGAX	OLFR1160			GM10030
	CD163L1	GM23237			GM26521
	VMN1R167	1700036G14RIK			BC023105
	FFAR2	FCRL1			
	IFITM2	INSRR			
	IFITM3	GM9054			
	GM7676	LPAR3			
	IRX3	IL21			
	GM24166	HOOK1			
	CCR1	ISG15			
	NHSL2	IDUA			
	GM8817	ATP8A1			
	GM36099	CXCL10			
		GBP8			
		GBP9			
		GBP4			
		GBP10			
		CD27			
		TAS2R104			
		TMEM86A			
		AU018091			
		VMN1R175			
		GM22986			
		PPP1R3FOS			
		MIR880			
		BC023105			

^*a*^ Shared genes between KLRG1 hi and lo cells are highlighted.

## DISCUSSION

There are multiple potential sources of IL-27 during inflammation that include DCs (3), macrophages (31), B cells (32), a subset of CD4^+^ T cells that produce IL-27 during murine malaria (33), and a newly described T_reg_ cell subset in the gut (34). Consistent with a previous report (35), the studies presented here identify a subset of activated monocytes as the major source of IL-27 during toxoplasmosis. While the anti-microbial activities of monocytes and monocyte-derived macrophages have an important role in resistance to *T. gondii* (36–38), there is a subset of monocytes that produce the immunosuppressive molecules IL-10 and PGE2 during this infection (39). In contrast to parasite control, these monocytes appear to contribute to tissue repair and a return to homeostasis. Early, NK cell-derived production of IFN-γ in the bone marrow shapes the development of these regulatory monocytes (39), but whether this process is also relevant to the generation of IL-27^+^ monocytes is unclear. While systemic levels of IL-27 decrease after the acute phase of infection, at later time points, when low-level parasite replication continues in the lungs and CNS, monocytes persist here in their production of IL-27. This is consistent with a model in which ongoing inflammation in the tissues sustains local IL-27 production to limit tissue damage. Nevertheless, while our previous studies highlighted the suppressive effects of IL-27 on T cells to limit immunopathology during toxoplasmosis (8), the detection of a transcriptional signature of enhanced innate responses at 5 dpi in the absence of IL-27 was unanticipated. There is a literature that highlights the impact of IL-27 on innate responses (40) and in a model of cecal ligation and puncture, blockade of IL-27 resulted in improved control of bacteria in the peritoneum and prevented the development of sepsis (13). Similarly, mice challenged with influenza in the absence of the IL-27R had an acute expansion of pathological neutrophils (41). IL-27 has also been shown to modify neutrophil maturation in the bone marrow, suppressing their production of pro-inflammatory cytokines while increasing their production of iron-scavenging molecules (42). In mice, monocytes and neutrophils do not express the IL-27R, but hematopoietic stem cells do and IL-27 can act on HSC to induce myelopoiesis and differentiation (43–45). Thus, it is possible that the absence of the regulatory effects of IL-27 on HSCs could impact on infection-induced emergency myelopoiesis.

Multiple studies using IL-27-deficient mice have highlighted that during infection IL-27 acts to limit a variety of Th1, Th2, and Th17 responses, and in its total absence, this can result in elevated levels of T cell-mediated collateral damage (5, 8, 15, 46–49). However, with reports of altered T cell homeostasis in the IL-27 KO mice (12, 50), it was unclear if this basal alteration contributes to the enhanced T cell responses observed in these mice during infection. That IL-27 neutralization in wild-type mice infected with *T. gondii* recapitulated the enhanced T cell responses, and immunopathology observed in IL-27R or IL-27p28-deficient mice supports the conclusion that IL-27 acts to limit pathological T cell activities. Nevertheless, questions remain about the basis for the CD4^+^ T cell-mediated immune pathology observed in the absence of IL-27. The finding that CD4 depletion resulted in a profound reduction in the TNFα^+^ monocytes at the peak of inflammation suggests a model in which elevated CD4^+^ T cell production of IFN-γ drives a population of monocytes that, in turn, contribute to tissue damage. Indeed, the shared profile with monocytes associated with sepsis would support this idea. Additionally, a similar circuit has been seen during infection with African trypanosomes (51). Here, the authors were able to show that CD4^+^ T cells regulated the formation of inflammatory Tip-DCs and that inhibition of the development of these cells using a CCR2^−/−^ mouse resulted in protection from pathology. However, it has been difficult for us to achieve complete monocyte depletions in our system both due to the technical difficulties of efficient depletion in this infectious system and due to the difficulties in balancing depletion with the critical role of monocytes in parasite control.

Another open question revolves around how IL-27 restrains the development of pathological T cells during toxoplasmosis. In this experimental system, minimally-differentiated, memory CD8^+^ T cells are CXCR3^+^ KLRG1^−^ and give rise to an intermediate CXCR3^+^ KLRG1^+^ population which, in turn, downregulates CXCR3 when they become terminally differentiated effector cells (29). We have observed that when IL-27 is limited by either neutralizing antibody treatment or in gene-deletion systems, this transition is accelerated. This has also been seen during *P. burghei* infection, where the loss of IL-27 signaling resulted in an enhancement in the numbers of KLRG1^+^, terminally differentiated CD4^+^ T cells (52). Additionally, a recent study on visceral leishmaniasis concluded that in IL-27-deficient hosts, enhanced mitochondrial activation is associated with increased Th1 cell expansion and suggested that endogenous IL-27 limited T-cell glycolysis (53). While that study indicated that IL-27 could impose metabolic control as a regulatory gate on T cell effector transition, here blockade of IL-27 did not result in any transcriptional changes in CD4^+^ T cell metabolic genes. Indeed, despite the changes observed in numbers of the KLRG1 hi and lo populations during IL-27 blockade, profiling of these populations did not reveal any major differences in the overall transcriptional programs of these populations, suggesting that IL-27 regulates the same network in both T cell subsets. Instead, both subsets have enhanced T_int_ phenotypes, with the KLRG1^lo^ cells having a corresponding loss of the memory precursor transcriptional program. This result suggests that in the absence of IL-27, the function and cellular identity of these populations has remained limited to the intermediate phenotype. Perhaps, the ability of IL-27 to maintain T cells in the memory precursor population restrains the expansion of the pathological effectors and thereby helps limit T cell-mediated pathology. Importantly, when IL-27 blockade was initiated during the chronic phase of infection, the magnitude of antigen-specific CD4^+^ and CD8^+^ T cell responses in the brain (the site of parasite persistence and inflammation) was enhanced while it remained unchanged in the periphery. This would be consistent with a model in which sustained production of IL-27 at sites of inflammation acts to continuously tune and shape these effector responses. Taken together, these studies have highlighted new questions about how early IL-27 impacts innate monocyte/macrophage responses, as well as CD4^+^ T cell responses, and how the intersection of these may contribute to pathology.

## SUPPLEMENTAL MATERIAL

Supplemental Figures mbio.00083-24-s0001.pdf Figures S1-S4.

ASM does not own the copyrights to Supplemental Material that may be linked to, or accessed through, an article. The authors have granted ASM a non-exclusive, world-wide license to publish the Supplemental Material files. Please contact the corresponding author directly for reuse.

## References

[1] Pflanz S, Timans JC, Cheung J, Rosales R, Kanzler H, Gilbert J, Hibbert L, Churakova T, Travis M, Vaisberg E, Blumenschein WM, Mattson JD, Wagner JL, To W, Zurawski S, McClanahan TK, Gorman DM, Bazan JF, de Waal Malefyt R, Rennick D, Kastelein RA. 2002. IL-27, a heterodimeric cytokine composed of EBI3 and p28 protein, induces proliferation of naive CD4^+^ T cells. Immunity 16:779–790. doi:10.1016/s1074-7613(02)00324-2 12121660

[2] Pflanz S, Hibbert L, Mattson J, Rosales R, Vaisberg E, Bazan JF, Phillips JH, McClanahan TK, de Waal Malefyt R, Kastelein RA. 2004. WSX-1 and glycoprotein 130 constitute a signal-transducing receptor for IL-27. J Immunol 172:2225–2231. doi:10.4049/jimmunol.172.4.2225 14764690

[3] Kilgore AM, Welsh S, Cheney EE, Chitrakar A, Blain TJ, Kedl BJ, Hunter CA, Pennock ND, Kedl RM. 2018. IL-27P28 production by XCR1^+^ dendritic cells and monocytes effectively predicts adjuvant-elicited CD8^+^ T cell responses. Immunohorizons 2:1–11. doi:10.4049/immunohorizons.1700054 29354801 PMC5771264

[4] Pennock ND, Gapin L, Kedl RM. 2014. IL-27 is required for shaping the magnitude, affinity distribution, and memory of T cells responding to subunit immunization. Proc Natl Acad Sci U S A 111:16472–16477. doi:10.1073/pnas.1407393111 25267651 PMC4246334

[5] Artis D, Villarino A, Silverman M, He W, Thornton EM, Mu S, Summer S, Covey TM, Huang E, Yoshida H, Koretzky G, Goldschmidt M, Wu GD, de Sauvage F, Miller HRP, Saris CJM, Scott P, Hunter CA. 2004. The IL-27 receptor (WSX-1) is an inhibitor of innate and adaptive elements of type 2 immunity. J Immunol 173:5626–5634. doi:10.4049/jimmunol.173.9.5626 15494513

[6] Hamano S, Himeno K, Miyazaki Y, Ishii K, Yamanaka A, Takeda A, Zhang M, Hisaeda H, Mak TW, Yoshimura A, Yoshida H. 2003. WSX-1 is required for resistance to Trypanosoma cruzi infection by regulation of proinflammatory cytokine production. Immunity 19:657–667. doi:10.1016/s1074-7613(03)00298-x 14614853

[7] Findlay EG, Greig R, Stumhofer JS, Hafalla JCR, de Souza JB, Saris CJ, Hunter CA, Riley EM, Couper KN. 2010. Essential role for IL-27 receptor signaling in prevention of Th1-mediated immunopathology during malaria infection. J Immunol 185:2482–2492. doi:10.4049/jimmunol.0904019 20631310

[8] Villarino A, Hibbert L, Lieberman L, Wilson E, Mak T, Yoshida H, Kastelein RA, Saris C, Hunter CA. 2003. The IL-27R (WSX-1) is required to suppress T cell hyperactivity during infection. Immunity 19:645–655. doi:10.1016/s1074-7613(03)00300-5 14614852

[9] Yoshida H, Hunter CA. 2015. The immunobiology of interleukin-27. Annu Rev Immunol 33:417–443. doi:10.1146/annurev-immunol-032414-112134 25861977

[10] Do J, Kim D, Kim S, Valentin-Torres A, Dvorina N, Jang E, Nagarajavel V, DeSilva TM, Li X, Ting AH, Vignali DAA, Stohlman SA, Baldwin WM, Min B. 2017. Treg-specific IL-27Rα deletion uncovers a key role for IL-27 in Treg function to control autoimmunity. Proc Natl Acad Sci U S A 114:10190–10195. doi:10.1073/pnas.1703100114 28874534 PMC5617261

[11] Zhang S, Liang R, Luo W, Liu C, Wu X, Gao Y, Hao J, Cao G, Chen X, Wei J, Xia S, Li Z, Wen T, Wu Y, Zhou X, Wang P, Zhao L, Wu Z, Xiong S, Gao X, Gao X, Chen Y, Ge Q, Tian Z, Yin Z. 2013. High susceptibility to liver injury in IL-27 p28 conditional knockout mice involves intrinsic interferon-γ dysregulation of CD4^+^ T cells. Hepatology 57:1620–1631. doi:10.1002/hep.26166 23175475

[12] Ahmadi F, Junghus F, Ashworth C, Lappalainen A, Mörbe U, Kotarsky K, Agace WW. 2023. cDC1-derived IL-27 regulates small intestinal CD4^+^ T cell homeostasis in mice. J Exp Med 220:e20221090. doi:10.1084/jem.20221090 36515659 PMC9754766

[13] Wirtz S, Tubbe I, Galle PR, Schild HJ, Birkenbach M, Blumberg RS, Neurath MF. 2006. Protection from lethal septic peritonitis by neutralizing the biological function of interleukin 27. J Exp Med 203:1875–1881. doi:10.1084/jem.20060471 16880260 PMC2118378

[14] Yarovinsky F. 2014. Innate immunity to Toxoplasma gondii infection. Nat Rev Immunol 14:109–121. doi:10.1038/nri3598 24457485

[15] Stumhofer JS, Laurence A, Wilson EH, Huang E, Tato CM, Johnson LM, Villarino AV, Huang Q, Yoshimura A, Sehy D, Saris CJM, O’Shea JJ, Hennighausen L, Ernst M, Hunter CA. 2006. Interleukin 27 negatively regulates the development of interleukin 17–producing T helper cells during chronic inflammation of the central nervous system. Nat Immunol 7:937–945. doi:10.1038/ni1376 16906166

[16] Aghayev T, Mazitova AM, Fang JR, Peshkova IO, Rausch M, Hung M, White KF, Masia R, Titerina EK, Fatkhullina AR, et al.. 2022. Il27 signaling serves as an immunologic checkpoint for innate cytotoxic cells to promote hepatocellular carcinoma. Cancer Discov 12:1960–1983. doi:10.1158/2159-8290.CD-20-1628 35723626 PMC9357073

[17] Park J, DeLong JH, Knox JJ, Konradt C, Wojno EDT, Hunter CA, Bäumler AJ. 2019. Impact of interleukin-27p28 on T and B cell responses during toxoplasmosis. Infect Immun 87:e00455-19. doi:10.1128/IAI.00455-19 31548322 PMC6867838

[18] Irizarry RA, Hobbs B, Collin F, Beazer-Barclay YD, Antonellis KJ, Scherf U, Speed TP. 2003. Exploration, normalization, and summaries of high density oligonucleotide array probe level data. Biostatistics 4:249–264. doi:10.1093/biostatistics/4.2.249 12925520

[19] Alidzanovic L, Starlinger P, Schauer D, Maier T, Feldman A, Buchberger E, Stift J, Koeck U, Pop L, Gruenberger B, Gruenberger T, Brostjan C. 2016. The VEGF rise in blood of bevacizumab patients is not based on tumor escape but a host-blockade of VEGF clearance. Oncotarget 7:57197–57212. doi:10.18632/oncotarget.11084 27527865 PMC5302983

[20] Lachmann N, Terasaki PI, Budde K, Liefeldt L, Kahl A, Reinke P, Pratschke J, Rudolph B, Schmidt D, Salama A, Schönemann C. 2009. Anti-human leukocyte antigen and donor-specific antibodies detected by luminex posttransplant serve as biomarkers for chronic rejection of renal allografts. Transplantation 87:1505–1513. doi:10.1097/TP.0b013e3181a44206 19461487

[21] Ozer J, Ratner M, Shaw M, Bailey W, Schomaker S. 2008. The current state of serum biomarkers of hepatotoxicity. Toxicology 245:194–205. doi:10.1016/j.tox.2007.11.021 18291570

[22] Hall AO, Beiting DP, Tato C, John B, Oldenhove G, Lombana CG, Pritchard GH, Silver JS, Bouladoux N, Stumhofer JS, Harris TH, Grainger J, Wojno EDT, Wagage S, Roos DS, Scott P, Turka LA, Cherry S, Reiner SL, Cua D, Belkaid Y, Elloso MM, Hunter CA. 2012. The cytokines interleukin 27 and interferon-γ promote distinct treg cell populations required to limit infection-induced pathology. Immunity 37:511–523. doi:10.1016/j.immuni.2012.06.014 22981537 PMC3477519

[23] Newman AM, Liu CL, Green MR, Gentles AJ, Feng W, Xu Y, Hoang CD, Diehn M, Alizadeh AA. 2015. Robust enumeration of cell subsets from tissue expression profiles. Nat Methods 12:453–457. doi:10.1038/nmeth.3337 25822800 PMC4739640

[24] Reyes M, Filbin MR, Bhattacharyya RP, Billman K, Eisenhaure T, Hung DT, Levy BD, Baron RM, Blainey PC, Goldberg MB, Hacohen N. 2020. An immune-cell signature of bacterial sepsis. Nat Med 26:333–340. doi:10.1038/s41591-020-0752-4 32066974 PMC7235950

[25] Hatton RD, Harrington LE, Luther RJ, Wakefield T, Janowski KM, Oliver JR, Lallone RL, Murphy KM, Weaver CT. 2006. A distal conserved sequence element controls Ifng gene expression by T cells and NK cells. Immunity 25:717–729. doi:10.1016/j.immuni.2006.09.007 17070076

[26] Clark JT, Weizman O-E, Aldridge DL, Shallberg LA, Eberhard J, Lanzar Z, Wasche D, Huck JD, Zhou T, Ring AM, Hunter CA. 2023. IL-18BP mediates the balance between protective and pathological immune responses to Toxoplasma gondii. Cell Rep 42:112147. doi:10.1016/j.celrep.2023.112147 36827187 PMC10131179

[27] Grover HS, Blanchard N, Gonzalez F, Chan S, Robey EA, Shastri N. 2012. The Toxoplasma gondii peptide AS15 elicits CD4 T cells that can control parasite burden. Infect Immun 80:3279–3288. doi:10.1128/IAI.00425-12 22778097 PMC3418726

[28] Wilson DC, Grotenbreg GM, Liu K, Zhao Y, Frickel E-M, Gubbels M-J, Ploegh HL, Yap GS, Denkers EY. 2010. Differential regulation of effector- and central-memory responses to Toxoplasma gondii infection by IL-12 revealed by tracking of Tgd057-specific CD8+ T cells. PLoS Pathog 6:e1000815. doi:10.1371/journal.ppat.1000815 20333242 PMC2841619

[29] Chu HH, Chan S-W, Gosling JP, Blanchard N, Tsitsiklis A, Lythe G, Shastri N, Molina-París C, Robey EA. 2016. Continuous effector CD8^+^ T cell production in a controlled persistent infection is sustained by a proliferative intermediate population. Immunity 45:159–171. doi:10.1016/j.immuni.2016.06.013 27421704 PMC4956557

[30] Huang H, Zhou P, Wei J, Long L, Shi H, Dhungana Y, Chapman NM, Fu G, Saravia J, Raynor JL, Liu S, Palacios G, Wang Y-D, Qian C, Yu J, Chi H. 2021. In vivo CRISPR screening reveals nutrient signaling processes underpinning CD8^+^ T cell fate decisions. Cell 184:1245–1261. doi:10.1016/j.cell.2021.02.021 33636132 PMC8101447

[31] Hause L, Al-Salleeh FM, Petro TM. 2007. Expression of IL-27 p28 by Theiler’s virus-infected macrophages depends on TLR3 and TLR7 activation of JNK-MAP-kinases. Antiviral Res 76:159–167. doi:10.1016/j.antiviral.2007.06.013 17675254

[32] Klarquist J, Cross EW, Thompson SB, Willett B, Aldridge DL, Caffrey-Carr AK, Xu Z, Hunter CA, Getahun A, Kedl RM. 2021. B cells promote CD8 T cell primary and memory responses to subunit vaccines. Cell Rep 36:109591. doi:10.1016/j.celrep.2021.109591 34433030 PMC8456706

[33] Kimura D, Miyakoda M, Kimura K, Honma K, Hara H, Yoshida H, Yui K. 2016. Interleukin-27-producing CD4^+^ T cells regulate protective immunity during malaria parasite infection. Immunity 44:672–682. doi:10.1016/j.immuni.2016.02.011 26968425

[34] Lin C-H, Wu C-J, Cho S, Patkar R, Huth WJ, Lin L-L, Chen M-C, Israelsson E, Betts J, Niedzielska M, Patel SA, Duong HG, Gerner RR, Hsu C-Y, Catley M, Maciewicz RA, Chu H, Raffatellu M, Chang JT, Lu L-F. 2023. Selective IL-27 production by intestinal regulatory T cells permits gut-specific regulation of TH17 cell immunity. Nat Immunol 24:2108–2120. doi:10.1038/s41590-023-01667-y 37932457 PMC11058069

[35] Detavernier A, Azouz A, Shehade H, Splittgerber M, Van Maele L, Nguyen M, Thomas S, Achouri Y, Svec D, Calonne E, Fuks F, Oldenhove G, Goriely S. 2019. Monocytes undergo multi-step differentiation in mice during oral infection by Toxoplasma gondii. Commun Biol 2:472. doi:10.1038/s42003-019-0718-6 31872076 PMC6920430

[36] Dunay IR, Damatta RA, Fux B, Presti R, Greco S, Colonna M, Sibley LD. 2008. Gr1^+^ inflammatory monocytes are required for mucosal resistance to the pathogen Toxoplasma gondii. Immunity 29:306–317. doi:10.1016/j.immuni.2008.05.019 18691912 PMC2605393

[37] Egan CE, Sukhumavasi W, Bierly AL, Denkers EY. 2008. Understanding the multiple functions of Gr-1^+^ cell subpopulations during microbial infection. Immunol Res 40:35–48. doi:10.1007/s12026-007-0061-8 18193362

[38] Goldszmid RS, Caspar P, Rivollier A, White S, Dzutsev A, Hieny S, Kelsall B, Trinchieri G, Sher A. 2012. NK cell-derived interferon-γ orchestrates cellular dynamics and the differentiation of monocytes into dendritic cells at the site of infection. Immunity 36:1047–1059. doi:10.1016/j.immuni.2012.03.026 22749354 PMC3412151

[39] Askenase MH, Han S-J, Byrd AL, Morais da Fonseca D, Bouladoux N, Wilhelm C, Konkel JE, Hand TW, Lacerda-Queiroz N, Su X, Trinchieri G, Grainger JR, Belkaid Y. 2015. Bone-marrow-resident NK cells prime monocytes for regulatory function during infection. Immunity 42:1130–1142. doi:10.1016/j.immuni.2015.05.011 26070484 PMC4472558

[40] Passos ST, Silver JS, O’Hara AC, Sehy D, Stumhofer JS, Hunter CA. 2010. IL-6 promotes NK cell production of IL-17 during toxoplasmosis. J Immunol 184:1776–1783. doi:10.4049/jimmunol.0901843 20083665 PMC3757499

[41] Liu FDM, Kenngott EE, Schröter MF, Kühl A, Jennrich S, Watzlawick R, Hoffmann U, Wolff T, Norley S, Scheffold A, Stumhofer JS, Saris CJM, Schwab JM, Hunter CA, Debes GF, Hamann A. 2014. Timed action of IL-27 protects from immunopathology while preserving defense in influenza. PLoS Pathog 10:e1004110. doi:10.1371/journal.ppat.1004110 24809349 PMC4014457

[42] Zhao X, Ting S-M, Liu C-H, Sun G, Kruzel M, Roy-O’Reilly M, Aronowski J. 2017. Neutrophil polarization by IL-27 as a therapeutic target for intracerebral hemorrhage. Nat Commun 8:602. doi:10.1038/s41467-017-00770-7 28928459 PMC5605643

[43] Seita J, Asakawa M, Ooehara J, Takayanagi S-I, Morita Y, Watanabe N, Fujita K, Kudo M, Mizuguchi J, Ema H, Nakauchi H, Yoshimoto T. 2008. Interleukin-27 directly induces differentiation in hematopoietic stem cells. Blood 111:1903–1912. doi:10.1182/blood-2007-06-093328 18042804

[44] Peshkova IO, Aghayev T, Fatkhullina AR, Makhov P, Titerina EK, Eguchi S, Tan YF, Kossenkov AV, Khoreva MV, Gankovskaya LV, Sykes SM, Koltsova EK. 2019. IL-27 receptor-regulated stress myelopoiesis drives abdominal aortic aneurysm development. Nat Commun 10:5046. doi:10.1038/s41467-019-13017-4 31695038 PMC6834661

[45] Furusawa J, Mizoguchi I, Chiba Y, Hisada M, Kobayashi F, Yoshida H, Nakae S, Tsuchida A, Matsumoto T, Ema H, Mizuguchi J, Yoshimoto T. 2016. Promotion of expansion and differentiation of hematopoietic stem cells by interleukin-27 into myeloid progenitors to control infection in emergency myelopoiesis. PLoS Pathog 12:e1005507. doi:10.1371/journal.ppat.1005507 26991425 PMC4798290

[46] Anderson CF, Stumhofer JS, Hunter CA, Sacks D. 2009. IL-27 regulates IL-10 and IL-17 from CD4^+^ cells in nonhealing Leishmania major infection. J Immunol 183:4619–4627. doi:10.4049/jimmunol.0804024 19748991 PMC2749572

[47] Yoshimoto T, Yoshimoto T, Yasuda K, Mizuguchi J, Nakanishi K. 2007. IL-27 suppresses Th2 cell development and Th2 cytokines production from polarized Th2 cells: a novel therapeutic way for Th2-mediated allergic inflammation. J Immunol 179:4415–4423. doi:10.4049/jimmunol.179.7.4415 17878337

[48] Quirino GFS, Nascimento MSL, Davoli-Ferreira M, Sacramento LA, Lima MHF, Almeida RP, Carregaro V, Silva JS. 2016. Interleukin-27 (IL-27) mediates susceptibility to visceral leishmaniasis by suppressing the IL-17–neutrophil response. Infect Immun 84:2289–2298. doi:10.1128/IAI.00283-16 27245409 PMC4962641

[49] Young A, Linehan E, Hams E, O’Hara Hall AC, McClurg A, Johnston JA, Hunter CA, Fallon PG, Fitzgerald DC. 2012. Cutting edge: suppression of GM-CSF expression in murine and human T cells by IL-27. J Immunol 189:2079–2083. doi:10.4049/jimmunol.1200131 22837488 PMC3424384

[50] Lin C-H, Chen M-C, Lin L-L, Christian DA, Min B, Hunter CA, Lu L-F. 2021. Gut epithelial IL-27 confers intestinal immunity through the induction of intraepithelial lymphocytes. J Exp Med 218:e20210021. doi:10.1084/jem.20210021 34554189 PMC8480671

[51] Liu G, Abas O, Fu Y, Chen Y, Strickland AB, Sun D, Shi M, Sher A. 2021. IL-27 negatively regulates tip-DC development during infection. mBio 12:e03385-20. doi:10.1128/mBio.03385-20 33593983 PMC8545113

[52] Villegas-Mendez A, de Souza JB, Lavelle S-W, Gwyer Findlay E, Shaw TN, van Rooijen N, Saris CJ, Hunter CA, Riley EM, Couper KN. 2013. IL-27 receptor signalling restricts the formation of pathogenic, terminally differentiated Th1 cells during malaria infection by repressing IL-12 dependent signals. PLoS Pathog 9:e1003293. doi:10.1371/journal.ppat.1003293 23593003 PMC3623720

[53] Montes de Oca M, de Labastida Rivera F, Winterford C, Frame TCM, Ng SS, Amante FH, Edwards CL, Bukali L, Wang Y, Uzonna JE, Kuns RD, Zhang P, Kabat A, Klein Geltink RI, Pearce EJ, Hill GR, Engwerda CR, Scott P. 2020. IL-27 signalling regulates glycolysis in Th1 cells to limit immunopathology during infection. PLoS Pathog 16:e1008994. doi:10.1371/journal.ppat.1008994 33049000 PMC7584222

