# Supplementary material for: Endogenous IL-27 during toxoplasmosis limits early monocyte responses and their inflammatory activation by pathological T cells
Source: mBio. 2024 Feb 20;15(3):e00083-24. doi: 10.1128/mbio.00083-24 (PMC10936422; doi:10.1128/mbio.00083-24)
Supplement: Supplemental Figures — Figures S1-S4. [file mbio.00083-24-s0001.pdf]

Supplemental Figure 1

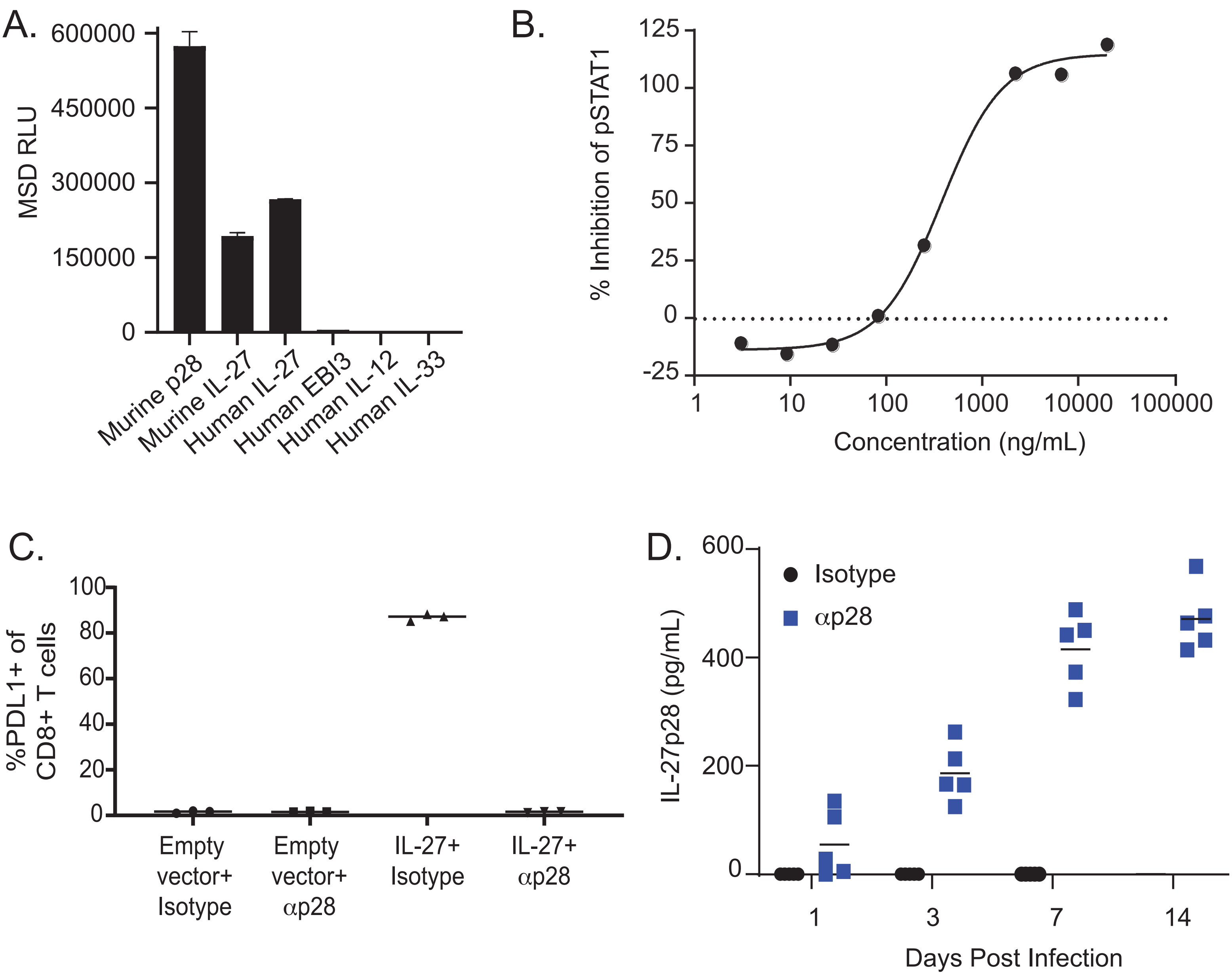

Supplemental Figure 2

A.

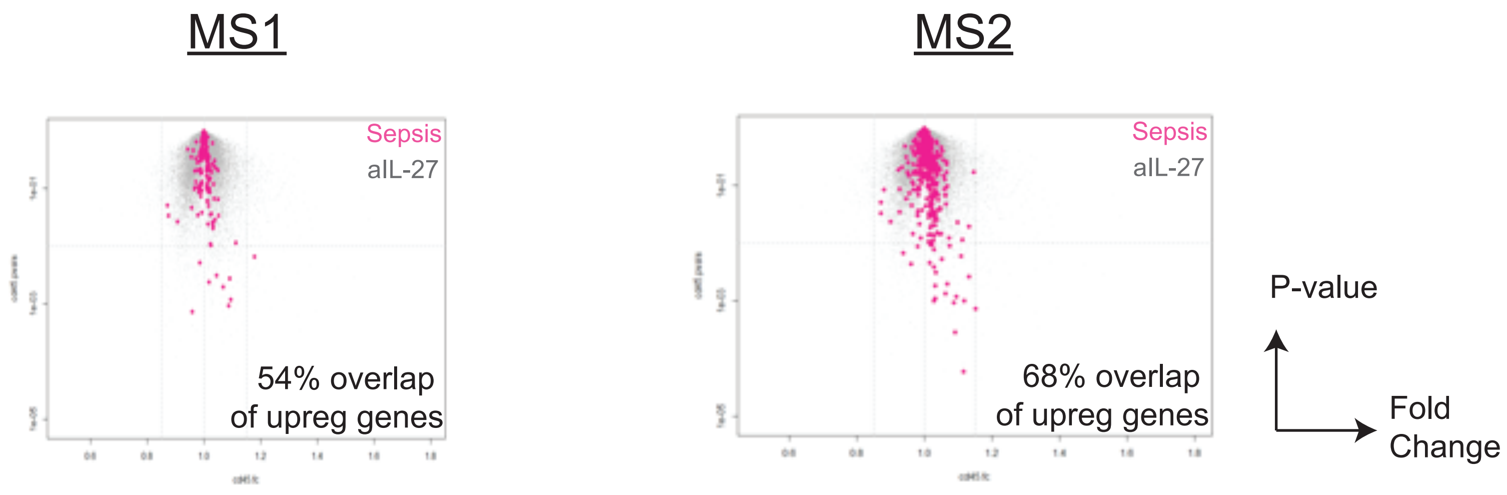

B.

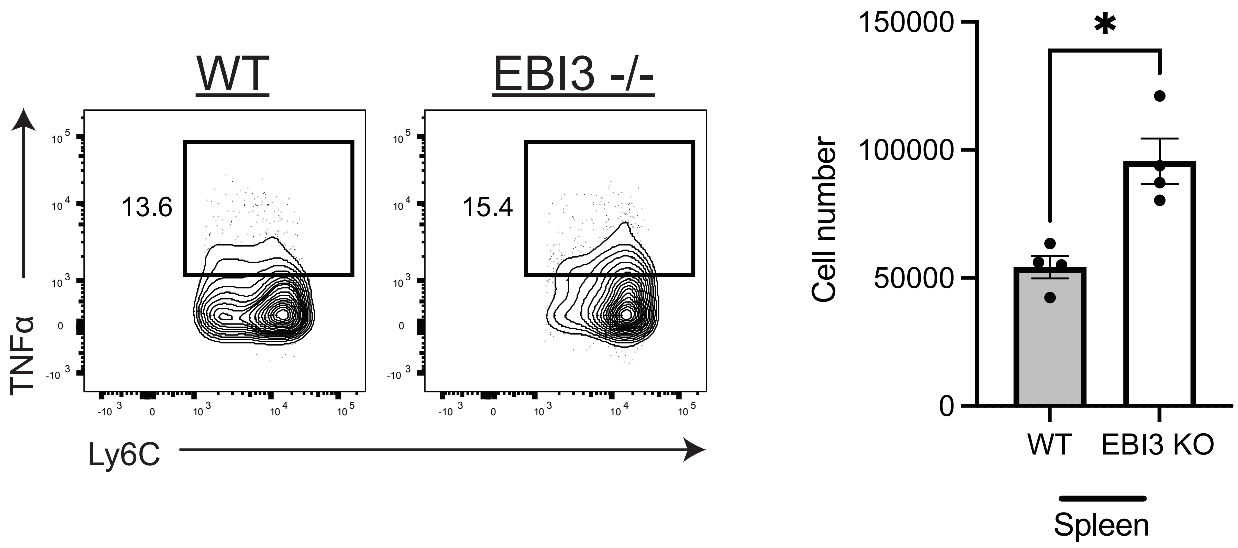

C.

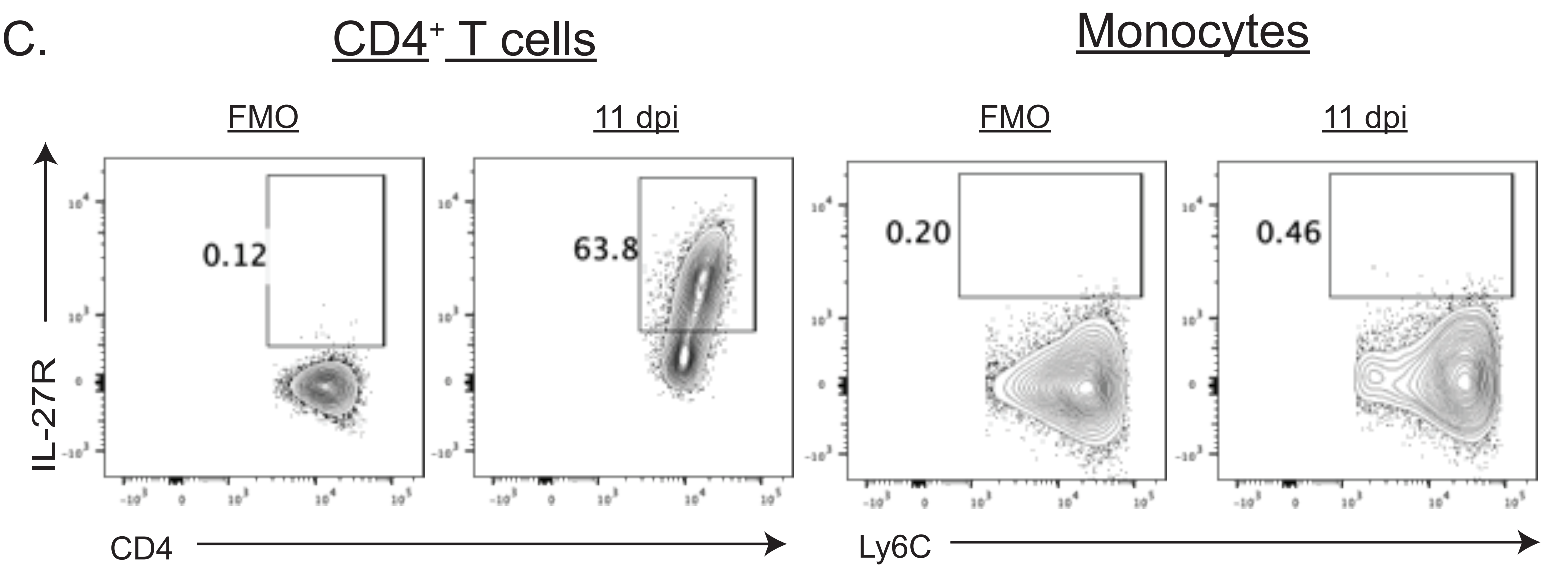

Supplemental Figure 3

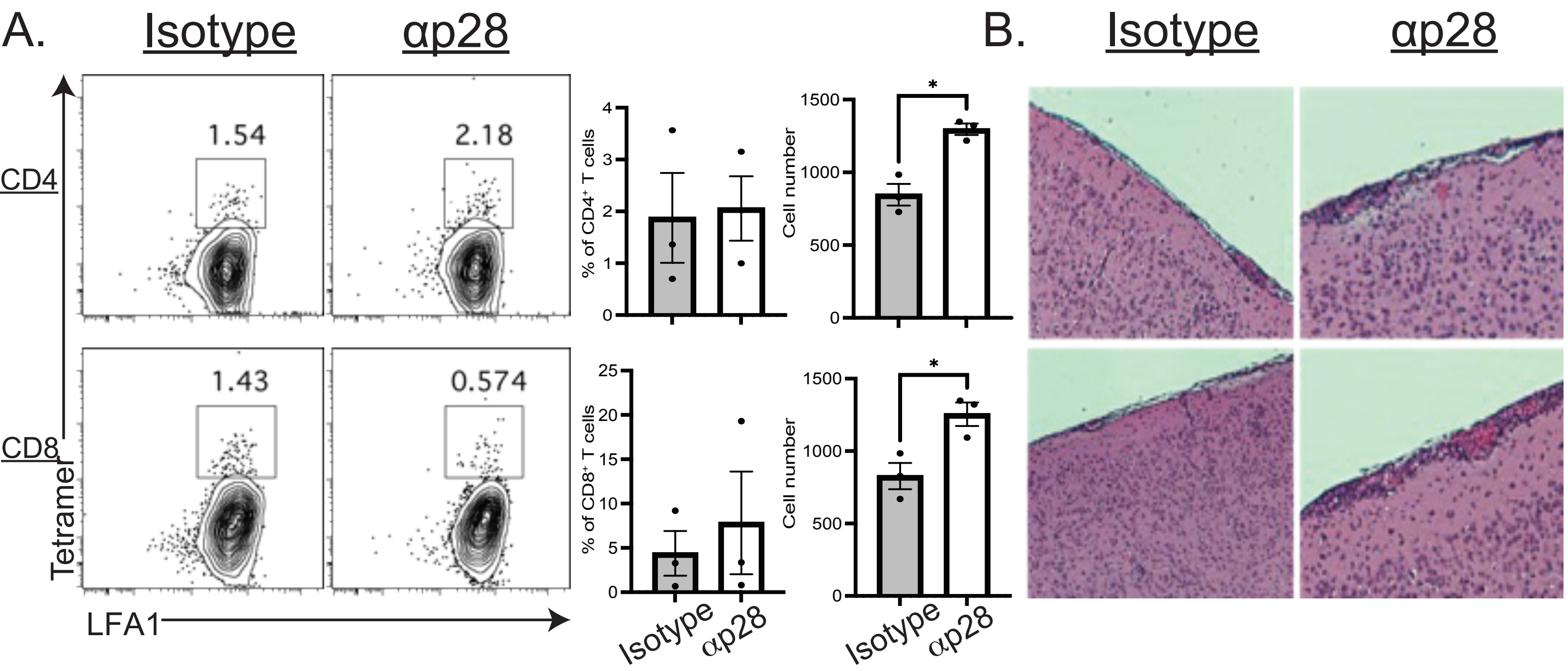

Supplemental Figure 4

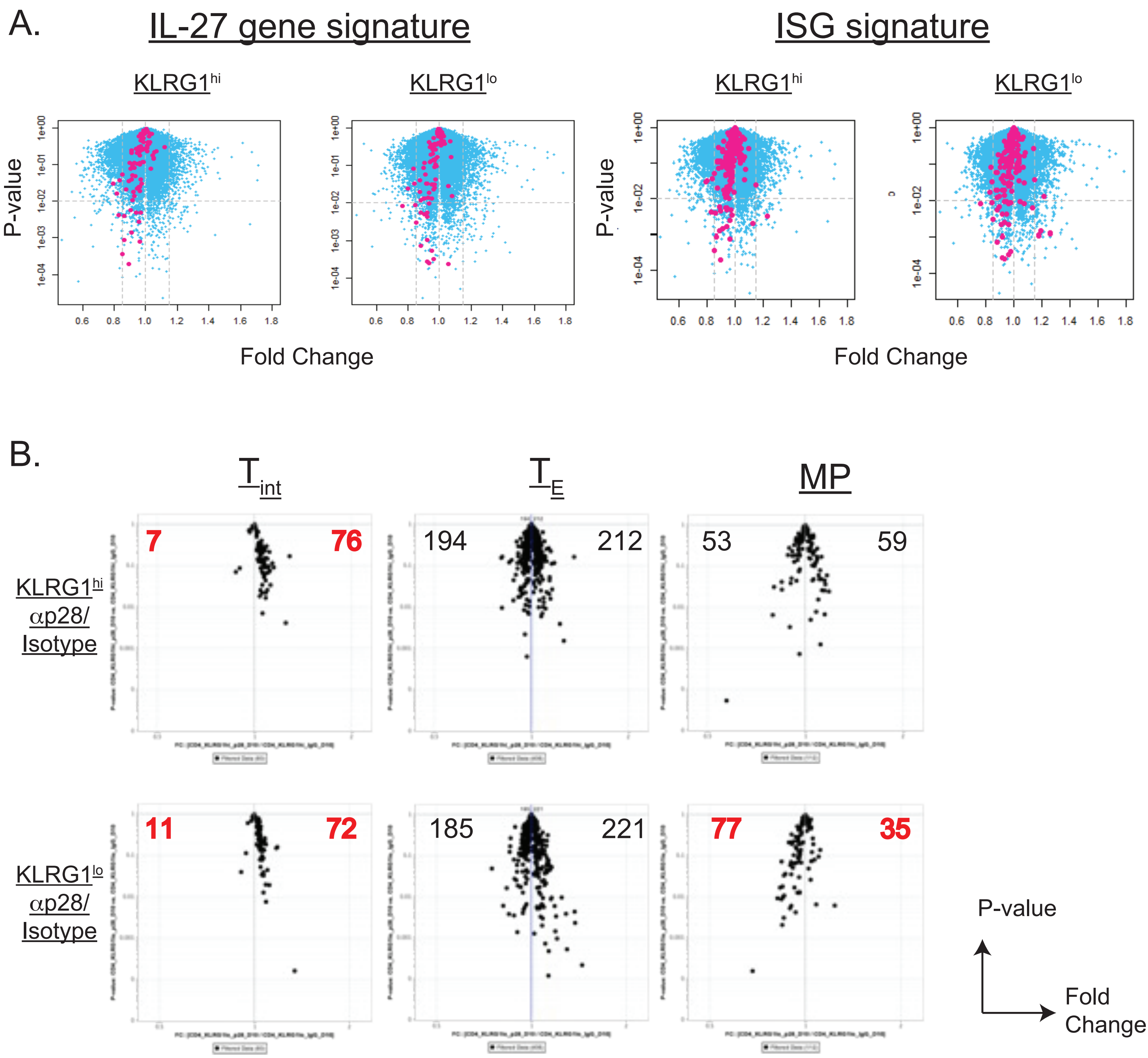

Supp. Fig. 1. Validation of IL-27p28 blockade.

SRF381 is a fully human IgG1 antibody that binds to human and murine IL-27, with specificity for the p28 subunit and blocks IL-27 signaling. (A) SRF381 binds to recombinant mouse IL-27, mouse p28, and human IL-27, but not recombinant human EBI3, human IL-12, and human IL-23 (all from R&D Systems) by MSD. (B) SRF381 inhibits recombinant murine IL-27 (20 ng/ml) induced pSTAT1 phosphorylation in splenic $CD3^+$ T cells in vitro by flow cytometry. The inhibition of pSTAT1 signaling by SRF381 was dose dependent with an IC50 of ~444 ng/ml. (C) SRF381 administration inhibits PD-L1 expression in $CD8^+$ T cells by flow cytometry after hydrodynamic injection of murine IL-27 minicircle DNA. (D) SRF381 administration leads to increased levels of murine p28 accumulation over time in the plasma of mice as detected by an anti-mouse IL-27 p28/IL-30 MSD.

Supp. Fig. 2. Monocytes are impacted early during toxoplasmosis by loss of IL-27 signaling. (A) The gene signature generated during IL-27p28 blockade at 5 dpi (Fig. 3) was compared to the gene signatures of various human monocyte subsets seen during sepsis (pink). The percentage of overlapping genes that were upregulated during blockade is shown. (B) WT and mice lacking the EBI3 subunit of IL-27 were infected with *T. gondii* and splenocytes analyzed at 5 dpi by flow cytometry for monocyte production of TNFα. Representative flow plots are shown (left) and number of expressing cells quantified (right). (C) IL-27Rα expression was measured on $CD4^+$ T cells ($CD19^-$, $CD3^+$, $CD4^+$; left panels) and monocytes ($CD19^-$, $CD3^-$, $CD11b^+$, $Ly6G^-$, $Ly6C^+$; right panels) at 11 dpi. Statistical analysis performed using Welch's T-test. * indicates $p \leq 0.05$.

Supp. Fig. 3. Immune response to anti-IL-27p28 antibody in chronic toxoplasmosis. (A) *T. gondii*-specific $CD4^+$ (top) and $CD8^+$ (bottom) T cells in the brain of isotype IgG and anti-IL-

27p28 antibody mice in chronic infection. Representative flow plots are shown (left) and the percentages and number of cells quantified (right). (B) Representative brain histology is observed using H&E staining. Magnification 10X. Representative and combined data collected (mean ± SEM, n=7-9) from 3 independent experiments. *Significant differences between indicated groups (P<0.05).

Supp. Fig. 4. Gene expression analysis of $CD4^+$ T cells during acute toxoplasmosis with IL-27p28 blockade. (A) Gene expression in $CD4^+$, $Tetramer^+$, $KLRG1^{hi/lo}$ T cells from the spleens of infected mice treated with IL-27p28 blocking antibody or isotype control at 10 dpi was measured. Expression of hallmark genes from a curated IL-27 gene signature (left plots) and ISG genes (right plots) was analyzed in these T cell subsets. Pink dots indicate expression of genes from the corresponding reference signatures. (B) $CD4^+$, $Tetramer^+$, $KLRG1^{hi/lo}$ T cells from above were further analyzed for their expression of genes associated with intermediate (T_{int}), effector (T_E), or memory progenitor (MP) T cell subsets (29). The expression of these genes in cells from blocking antibody treated vs isotype treated mice is shown, with genes in isotype treated cells shown on the left of the volcano plots and blocking antibody treated on the right. Bolded numbers on each plot indicate the numbers of genes from the indicated signatures that are either a fold-change of >1 (right side, higher in blocking antibody treated), or a fold-change of less <1 (left side, higher in isotype treated). Note, if there is no influence of IL-27 on the signature an approximately equivalent number of genes would be expected for a fold-change of >1 and <1.
